# Comparative cardiovascular safety of LABA/LAMA FDC versus LABA/ICS FDC in patients with chronic obstructive pulmonary disease: a population-based cohort study with a target trial emulation framework

**DOI:** 10.1186/s12931-023-02545-9

**Published:** 2023-09-29

**Authors:** Chun-Yu Chen, Sheng-Wei Pan, Chia-Chen Hsu, Jason J. Liu, Hiraku Kumamaru, Yaa-Hui Dong

**Affiliations:** 1 Institute of Public Health, School of Medicine, National Yang Ming Chiao Tung University, Taipei, Taiwan; 2 Department of Pharmacy, College of Pharmaceutical Sciences, National Yang Ming Chiao Tung University, Taipei, Taiwan; 3 Department of Chest Medicine, Taipei Veterans General Hospital, Taipei, Taiwan; 4 Faculty of Medicine, School of Medicine, National Yang Ming Chiao Tung University, Taipei, Taiwan; 5 Department of Pharmacy, Taipei Veterans General Hospital, Taipei, Taiwan; 6 Department of Healthcare Quality Assessment, Graduate School of Medicine, The University of Tokyo, Tokyo, Japan; 7 Institute of Hospital and Health Care Administration, School of Medicine, National Yang Ming Chiao Tung University, Taipei, Taiwan

**Keywords:** Chronic obstructive pulmonary disease, Long-acting β_2_ agonists/long-acting muscarinic antagonists (LABA/LAMA), Long-acting β_2_ agonists/inhaled corticosteroids (LABA/ICS), Fixed-dose combinations (FDC), Cardiovascular events, Cohort study, Target trial emulation framework

## Abstract

**Background:**

Use of combinations of long-acting β_2_ agonists/long-acting muscarinic antagonists (LABA/LAMA) in patients with chronic obstructive pulmonary disease (COPD) is increasing. Nevertheless, existing evidence on cardiovascular risk associated with LABA/LAMA versus another dual combination, LABA/inhaled corticosteroids (ICS), was limited and discrepant.

**Aim:**

The present cohort study aimed to examine comparative cardiovascular safety of LABA/LAMA and LABA/ICS with a target trial emulation framework, focusing on dual fixed-dose combination (FDC) therapies.

**Methods:**

We identified patients with COPD who initiated LABA/LAMA FDC or LABA/ICS FDC from a nationwide Taiwanese database during 2017–2020. The outcome of interest was a hospitalized composite cardiovascular events of acute myocardial infarction, unstable angina, heart failure, cardiac dysrhythmia, and ischemic stroke. Cox regression models were used to estimate hazard ratios (HRs) and 95% confidence intervals (CIs) for composite and individual cardiovascular events after matching up to five LABA/LAMA FDC initiators to one LABA/ICS FDC initiator using propensity scores (PS).

**Results:**

Among 75,926 PS-matched patients, use of LABA/LAMA FDC did not show a higher cardiovascular risk compared to use of LABA/ICS FDC, with a HR of 0.89 (95% CI, 0.78–1.01) for the composite events, 0.80 (95% CI, 0.61–1.05) for acute myocardial infarction, 1.48 (95% CI, 0.68–3.25) for unstable angina, 1.00 (95% CI, 0.80–1.24) for congestive heart failure, 0.62 (95% CI, 0.37–1.05) for cardiac dysrhythmia, and 0.82 (95% CI, 0.66–1.02) for ischemic stroke. The results did not vary substantially in several pre-specified sensitivity and subgroup analyses.

**Conclusion:**

Our findings provide important reassurance about comparative cardiovascular safety of LABA/LAMA FDC treatment among patients with COPD.

**Supplementary Information:**

The online version contains supplementary material available at 10.1186/s12931-023-02545-9.

## Introduction

Chronic obstructive pulmonary disease (COPD) poses a paramount clinical burden worldwide. It is the third and the eighth major cause of mortality globally and in Taiwan, respectively [1, 2]. Long-acting bronchodilators, including long-acting β_2_ agonists (LABA) and long-acting muscarinic antagonists (LAMA), are the central maintenance treatment in reducing COPD-related symptoms and exacerbations [3]. The combination therapy of LABA and LAMA (LABA/LAMA) is further indicated for patients with a high risk of acute exacerbations or with suboptimal response to LABA or LAMA monotherapy [3]. On the other hand, because of the pharmacological actions of LABA on β_2_ receptors and LAMA on M_3_ receptors [4, 5], sympathetic activation and potential cardiovascular risk of LABA and LAMA, especially as combination therapy, deserves paying attention.

One recent meta-analysis of clinical trials found that LABA/LAMA had a 42% higher risk of major adverse cardiovascular events (MACE) compared to another combination therapy of LABA and inhaled corticosteroids (ICS) (LABA/ICS) (risk ratio, 1.42; 95% confidence interval [CI], 1.11–1.81) [6]. Few real-world studies have evaluated cardiovascular risk of LABA/LAMA to LABA/ICS. One US cohort study did not observe an increased risk of hospitalized, composite cardiovascular events associated with LABA/LAMA (hazards ratio [HR], 0.85; 95% CI, 0.66–1.04) [7]. Another Taiwanese cohort study also did not show apparent risk of hospitalized, composite cardiovascular events associated with different LABA/LAMA (HRs ranging from 1.03 [95% CI, 0.83–1.29] to 1.29 [95% CI, 0.96–1.73]) [8].

There may be potential clinical and methodological issues accounting for the aforementioned discrepant findings. For example, the increased cardiovascular risk associated with LABA/LAMA found in the meta-analysis[6] was mainly driven by three efficacy trials in which patients had to experience exacerbation episodes and have apparent respiratory symptoms [9–11]. However, patients with clinically significant cardiovascular abnormalities were excluded. Moreover, the trial design of abrupt ICS withdrawal at randomization may exacerbate disease control and even cardiovascular outcomes for patients allocated to the LABA/LAMA treatment [12, 13]. The above cohort studies addressed the cardiovascular risk of LABA/LAMA in the daily practice; however, none of the studies specifically examined the risk among patients with cardiovascular disease or with longer treatment durations [7, 8].

With the availability and the increasing use of LABA/LAMA fixed-dose combination products (FDC, i.e., ≥ 2 active drugs in a single inhaler) in the market [14, 15], it becomes important to comprehensively assess cardiovascular safety associated with LABA/LAMA FDC use in the real-world settings. The present population-based cohort study aimed to examine comparative cardiovascular safety of LABA/LAMA FDC and LABA/ICS FDC in patients with COPD. LABA/ICS FDC, rather than LABA or LAMA monotherapy, was selected as an active comparison group since both LABA/LAMA FDC and LABA/ICS FDC are dual combination therapies and tend to be comparable in patient characteristics, which may mitigate potential confounding.

## Methods

### Data source

This present study used data from the Taiwan National Health Insurance Research Database (NHIRD), which included de-identified data of approximately 23 million beneficiaries enrolled in a single-payer national health insurance system [16, 17]. See Additional file 1: eMethods for detailed data source description.

### Cohort study with a target trial emulation framework

**Fig. 1:**
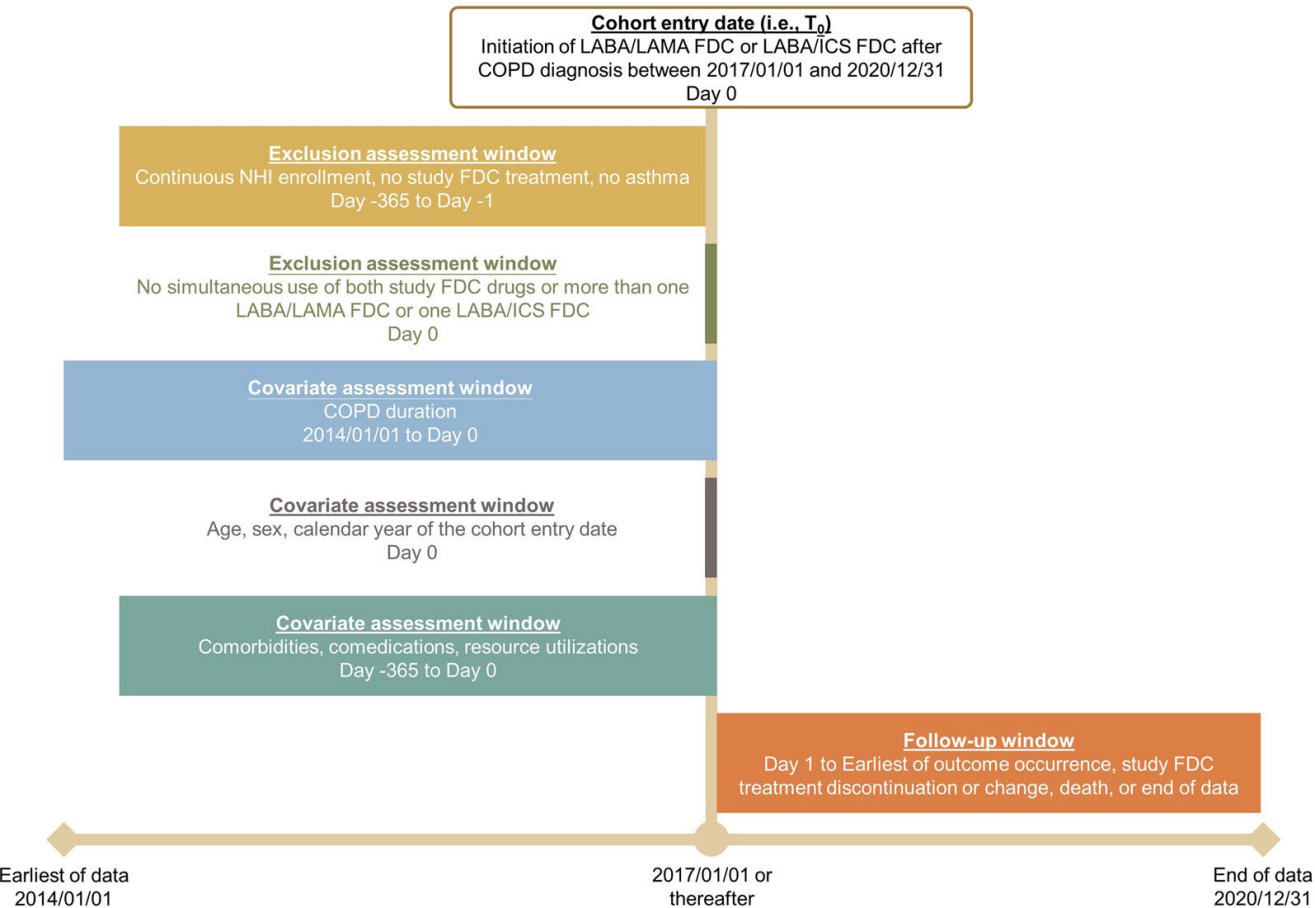
Graphic depiction of the cohort study design. *COPD* chronic obstructive pulmonary disease, *FDC* fixed-dose combinations, *ICS* inhaled corticosteroids, *LABA* long-acting β_2_ agonists, *LAMA* long-acting muscarinic antagonists, *NHI* National Health Insurance

To our knowledge, no existing trials aimed to examine cardiovascular safety issues of LABA/LAMA FDC versus LABA/ICS FDC among patients with COPD. Therefore, we specified components of a target trial (i.e., a hypothetical trial) and emulated the trial using Taiwan NHIRD. In another word, we conducted a cohort study with a target trial framework in which ascertainment of study population, exposure, outcomes, and baseline covariates were anchored at the cohort entry date (i.e., T_0_) and appropriate statistical analyses were applied to enhance causal inference estimation using real-world data [18–22]. See Additional file 1: Table S1 for specification of each component and Fig. 1 for the graphic depiction of the design. Corresponding approaches applied in the present study were also mentioned in our previous work [23, 24].

### Study population and exposure

Our study population comprised patients with COPD who initiated LABA/LAMA FDC or LABA/ICS FDC from the NHIRD between 2017/01/01 and 2020/12/31. COPD was defined as having ≥ 1 outpatient or inpatient relevant diagnosis code in any diagnosis position (see Additional file 1: Table S2 for codes [25]). Use of LABA/LAMA FDC or LABA/ICS FDC was defined based on outpatient pharmacy dispensing claims (see Additional file 1: Table S3 for codes). The cohort entry date (i.e., T_0_) was the date of the first dispensing of a LABA/LAMA FDC or LABA/ICS FDC after a diagnosis of COPD. Further exclusion criteria were presented in Additional file 1: eMethods.

### Study outcomes and follow-up

We defined the primary outcome, a composite of cardiovascular events, as the first hospitalization for acute myocardial infarction, unstable angina, congestive heart failure, cardiac dysrhythmia, or ischemic stroke. We ascertained each outcome based on inpatient diagnosis codes recorded in the primary positions. These claims-based algorithms have been shown to have positive predictive values of 76–100% (Additional file 1: Table S4) [26–35].

The primary analysis applied an on-treatment approach, which followed patients from cohort entry to outcome occurrence, study FDC treatment discontinuation or change, death, or end of data (2020/12/31), whichever came first. Study FDC treatment discontinuation was defined using a grace period of 60 days between the end date of one dispensing and the start date of the next dispensing, if any; and the discontinuation date was 60 days after the end of the last dispensing. Study FDC treatment change was defined as a dispensing of ICS-containing regimens or another LABA/LAMA FDC for LABA/LAMA FDC initiators, and a dispensing of LAMA-containing regimens or another LABA/ICS FDC for LABA/ICS FDC initiators.

### Positive control outcome

Trial and real-world data have shown that use of LABA/LAMA has a lower risk of pneumonia compared to use of LABA/ICS (relative risk of 0.57 [95% CI, 0.42–0.79] and 0.66 [95% CI, 0.50–0.87], respectively) [36, 37]. Therefore, we chose pneumonia as a positive control outcome and examined if our design could identify a decreased risk of pneumonia associated with LABA/LAMA FDC. This positive control outcome approach is commonly applied in observational studies for assessing internal validity [38, 39]. The pneumonia outcome was determined using inpatient diagnosis codes recorded in any positions, with the claims-based algorithm exhibiting a positive predictive value of 88% (Additional file 1: Table S4) [40].

### Covariates

We assessed > 80 potential baseline confounders including age at cohort entry, sex, COPD duration defined as the duration from the first recorded date of COPD diagnosis (looking back until 2014/01/01) to the cohort entry date, calendar year of the cohort entry date, as well as comorbidities (e.g., cardiovascular disease), other medication use (e.g., cardiovascular medication), influenza or pneumococcal vaccination, and outpatient visits or hospital admissions for any reasons, for COPD, or for cardiovascular disease that may be associated with both the use of study FDC treatment and the risks of study outcomes. These characteristics were determined using diagnosis files or pharmacy dispensing records derived from outpatient or inpatient claims of the NHIRD within 365 days before cohort entry. Additional file 1: Tables S5 and S6 present detailed covariate information.

### Statistical analysis

Using all above predefined, claims-based covariates, we estimated the baseline propensity scores (PS), i.e., the probabilities of initiating LABA/LAMA FDC versus LABA/ICS FDC, with a logistic regression model. Because there were many more LABA/LAMA FDC initiators, up to five LABA/LAMA FDC initiators were matched to one LABA/ICS FDC initiator using a nearest-neighbor algorithm without replacement and with a maximum matching caliper of 0.025 on the PS scale (see Additional file 1: eMethods for detailed variable-ratio matching steps) [41]. We computed standardized differences for each covariate to evaluate covariate distribution before and after PS matching, with a value < 0.1 in absolute value indicating balance between treatment groups [42].

The incidence rates and 95% CIs for LABA/LAMA FDC and LABA/ICS FDC initiators were estimated according to a Poisson distribution. The cumulative incidence plots were derived by one minus the Kaplan–Meier estimate (i.e., complement of the Kaplan–Meier survival function). Using conventional Cox proportional hazards models, we estimated HRs and 95% CIs of composite cardiovascular events comparing LABA/LAMA FDC with LABA/ICS FDC before and after PS matching. We also examined the risks for the individual components of the composite outcome. All the analyses after PS matching took the variable-ratio matching into consideration.

### Sensitivity analyses

For composite cardiovascular events, we conducted four pre-specified sensitivity analyses to examine the robustness of the results comparing LABA/LAMA FDC with LABA/ICS. First, we used an intention-to-treat approach which continued to follow patients regardless of study FDC treatment discontinuation or change during follow-up and censored patients at the first of outcome occurrence, death, or end of data. Second, to account for the influence of competing risk from overall death, we re-draw cumulative incidence plots and applied the Fine‐Gray subdistribution hazard model for subdistribution HR estimation, which remains patients who have previously experienced overall death in the risk sets (Additional file 1: eMethods) [43, 44]. Third, to mitigate potential unmeasured confounding, we conducted high-dimensional PS (hd-PS) estimation which identifies and includes additional 100 empirically-identified, claims-based covariates in the PS model (Additional file 1: eMethods) [45, 46].

Finally, besides controlling for claims-based covariates, we attempted to additionally adjust for several important clinical parameters captured from the Taiwan National Health Insurance (NHI) Laboratory Database and the Taiwan COPD pay-for-performance (P4P) Database, both of which were recently established nationwide databases under series of national health policies in Taiwan (see Additional file 1: eMethods for detailed database description [47, 48]). Specifically, we measured the following 11 clinical parameters potentially related to the risks of outcomes from outpatient and inpatient health encounter records in the NHI Laboratory Database or the COPD P4P Database within 365 days before cohort entry, including laboratory test results (eosinophil, c-reactive protein, low-density lipoprotein-cholesterol, glycated hemoglobin, and glomerular filtration rate [GFR] or estimated GFR), lung function test results (predicted post-dose forced expiratory volume in one second [FEV_1_], post-dose FEV_1_/forced vital capacity), COPD Assessment Test (CAT) score, systolic blood pressure, and health behavior (body mass index and smoking status) (Additional file 1: Table S7). Because not all the patients had information on these parameters, we conducted multiple imputation to handle missing data issues (Additional file 1: eMethods) [49–51].

### Subgroup analyses

For composite cardiovascular events, we also conducted three pre-specified subgroup analyses (1) to evaluate potential effect measure modification by patient characteristic (age, sex, COPD duration, history of hospitalized COPD exacerbations, and history of cardiovascular diseases); (2) to assess whether the association was different comparing individual LABA/LAMA FDC with individual LABA/ICS FDC; (3) and to explore potential treatment duration-response relation for LABA/LAMA FDC (1–90, 91–180, 181–365, and > 365 days). We re-estimated the PS and re-matched patients within each subgroup [41, 52, 53]. See Additional file 1: Table S8 for details of each analysis. Besides comparing the subgroup-specific effect estimates, we applied the Wald test to formally test if the results differ materially across patient subgroups, and a p-value < 0.05 was considered statistically significant.

## Results

### Eligible patients

A total of 99,506 eligible patients who initiated a LABA/LAMA FDC (n = 61,221) or a LABA/ICS FDC (n = 38,285) were included. Vilanterol/umeclidinium (VIL/UME, n = 26,606) was the most commonly used LABA/LAMA FDC, followed by olodaterol/tiotropium (OLO/TIO, n = 19,189) and indacaterol/glycopyrronium (IND/GLY, n = 15,426). Formoterol/budesonide (FOR/BUD, n = 10,351) and formoterol/beclomethasone (FOR/BEC, n = 10,311) were the two most frequently used LABA/ICS FDC, followed by salmeterol/fluticasone (SAL/FLU, n = 9,605), vilanterol/fluticasone (VIL/FLU, n = 7,584), and formoterol/fluticasone (FOR/FLU, n = 434) (Additional file 1: Fig. S1).

Before PS matching, LABA/LAMA FDC initiators were slightly older (mean age in years: 70 versus 69) and more likely to be male (male %: 85 versus 65) than LABA/ICS FDC initiators. LABA/LAMA FDC initiators also tended to have a history of lung cancer, receive LABA or LAMA monotherapy, and have more frequent outpatient visits for COPD than LABA/ICS FDC initiators. In contrast, LABA/LAMA FDC initiators were less likely to receive ICS than LABA/ICS FDC initiators. The PS model yielded a c-statistic of 0.790. After 5:1 variable-ratio matching, a total of 75,926 patients were included (76% of the study cohort, 80% of LABA/LAMA FDC initiators, and 71% of LABA/ICS FDC initiators). PS matching achieved balance in all baseline claims-based characteristics (Table 1; Additional file 1: Table S9, Fig. S2).

**Table 1:** Selected patient characteristics of the eligible cohort before and after PS matching

	Before PS matching (n = 99,506)			After PS matching (n = 75,926)		
	LABA/LAMA FDC	LABA/ICS FDC	Standardized difference	LABA/LAMA FDC	LABA/ICS FDC	Standardized difference
	n = 61,221	n = 38,285		n = 48,864	n = 27,062	
				n = 27,062^a^	n = 27,062^a^	
Demographics						
Age, years, mean (SD)	70.23 (11.48)	68.64 (12.71)	0.132	68.63 (11.68)	68.69 (12.50)	− 0.005
Male, n (%)	52,023 (84.98)	25,032 (65.38)	0.466	20,580 (76.05)	20,893 (77.20)	− 0.027
COPD duration, days, mean (SD)^b^	680.71 (730.58)	657.27 (706.95)	0.033	652.08 (725.58)	659.15 (708.78)	− 0.010
Comorbidities, n (%)						
Hypertension	35,598 (58.15)	22,453 (58.65)	− 0.010	15,607 (57.67)	15,629 (57.75)	− 0.002
Ischemic heart disease or angina	16,541 (27.02)	9965 (26.03)	0.022	6992 (25.84)	7037 (26.00)	− 0.004
Myocardial infarction	1991 (3.25)	1,133 (2.96)	0.017	769 (2.84)	757 (2.80)	0.003
Coronary revascularization	1388 (2.27)	819 (2.14)	0.009	578 (2.14)	574 (2.12)	0.001
Cardiac dysrhythmia	10,215 (16.69)	6135 (16.02)	0.018	4224 (15.61)	4198 (15.51)	0.003
Congestive heart failure	9932 (16.22)	6333 (16.54)	− 0.009	4113 (15.20)	4134 (15.28)	− 0.002
Cerebrovascular disease	9440 (15.42)	6141 (16.04)	− 0.017	4019 (14.85)	4107 (15.18)	− 0.009
Ischemic stroke	4638 (7.58)	2840 (7.42)	0.006	1906 (7.04)	1926 (7.12)	− 0.003
Hemorrhagic stroke	1220 (1.99)	947 (2.47)	− 0.033	562 (2.08)	564 (2.08)	− 0.001
Peripheral vascular disease	1828 (2.99)	1126 (2.94)	0.003	777 (2.87)	791 (2.92)	− 0.003
Diabetes mellitus	17,051 (27.85)	11,164 (29.16)	− 0.029	7605 (28.10)	7574 (27.99)	0.003
Hyperlipidemia	22,042 (36.00)	14,589 (38.11)	− 0.044	10,196 (37.68)	10,110 (37.36)	0.007
Pneumonia	9273 (15.15)	5927 (15.48)	− 0.009	3792 (14.01)	3817 (14.10)	− 0.003
Influenza	4047 (6.61)	2557 (6.68)	− 0.003	1762 (6.51)	1755 (6.49)	0.001
Acute bronchitis	29,994 (48.99)	19,821 (51.77)	− 0.056	13,451 (49.70)	13,480 (49.81)	− 0.002
Chronic kidney disease	9554 (15.61)	5549 (14.49)	0.031	3720 (13.75)	3804 (14.06)	− 0.009
Medication use, n (%)						
ACEI or ARB	26,268 (42.91)	16,658 (43.51)	− 0.012	11,572 (42.76)	11,635 (42.99)	− 0.005
Selective β_1_ blockers	13,683(22.35)	8,378(21.88)	0.011	5850 (21.62)	5861 (21.66)	− 0.001
Non-selective β_1_ blockers	9672 (15.80)	6041 (15.78)	0.001	4147 (15.32)	4183 (15.46)	− 0.004
Calcium channel blockers	24,041 (39.27)	14,861 (38.82)	0.009	10,176 (37.60)	10,206 (37.71)	− 0.002
Diuretics	17,530 (28.63)	11,038 (28.83)	− 0.004	7093 (26.21)	7189 (26.56)	− 0.008
Other anti-hypertensive agents	6,798 (11.10)	3,729 (9.74)	0.045	2632 (9.73)	2692 (9.95)	− 0.007
Nitrates	11,198 (18.29)	6,741 (17.61)	0.018	4656 (17.20)	4700 (17.37)	− 0.004
Anti-arrhythmic agents	5677 (9.27)	3304 (8.63)	0.023	2166 (8.00)	2199 (8.13)	− 0.004
Digoxin	1772 (2.89)	1178 (3.08)	− 0.011	724 (2.68)	734 (2.71)	− 0.002
Aspirin	18,324 (29.93)	10,865 (28.38)	0.034	7781 (28.75)	7774 (28.73)	0.001
Clopidogrel	5,889 (9.62)	3,565 (9.31)	0.011	2,390 (8.83)	2,423 (8.95)	− 0.004
Warfarin	1,186 (1.94)	723 (1.89)	0.004	477 (1.76)	458 (1.69)	0.005
Direct thrombin or factor Xa inhibitors	3298 (5.39)	1928 (5.04)	0.016	1302 (4.81)	1282 (4.74)	0.003
Statins	16,761 (27.38)	10,797 (28.20)	− 0.018	7471 (27.61)	7471 (27.61)	0
Fibrates	2452 (4.01)	1639 (4.28)	− 0.014	1173 (4.33)	1208 (4.46)	− 0.006
Insulin	6016 (9.83)	3726 (9.73)	0.003	2259 (8.35)	2332 (8.62)	− 0.010
Metformin	10,245 (16.73)	6639 (17.34)	− 0.016	4569 (16.88)	4550 (16.81)	0.002
Sulfonylurea	6437 (10.51)	3984 (10.41)	0.004	2786 (10.29)	2777 (10.26)	0.001
Glinides	1634 (2.67)	1090 (2.85)	− 0.011	652 (2.41)	681 (2.52)	− 0.007
Thiazolidinedione	1721 (2.81)	1024 (2.67)	0.008	715 (2.64)	721 (2.66)	− 0.001
Alpha-glucosidase inhibitors	1703 (2.78)	1046 (2.73)	0.003	702 (2.59)	699 (2.58)	0.001
Dipeptidyl peptidase-4 inhibitors	7013 (11.46)	4471 (11.68)	− 0.007	2988 (11.04)	3009 (11.12)	− 0.002
Sodium-glucose cotransporter 2 Inhibitors	1160 (1.89)	740 (1.93)	− 0.003	532 (1.97)	516 (1.91)	0.004
Glucagon-like peptide-1 receptor agonists	167 (0.27)	114 (0.30)	− 0.005	74 (0.27)	64 (0.24)	0.007
Inhaled short-acting bronchodilators	27,375 (44.72)	15,665 (40.92)	0.077	10,445 (38.60)	10,646 (39.34)	− 0.015
Inhaled long-acting bronchodilators	26,359 (43.06)	10,374 (27.10)	0.339	4141 (15.30)	4122 (15.23)	0.002
ICS	1764 (2.88)	8454 (22.08)	− 0.607	1763 (6.51)	1862 (6.88)	− 0.015
Systemic bronchodilators	44,762 (73.12)	28,903 (75.49)	− 0.054	20,554 (75.95)	20,549 (75.93)	< 0.001
Systemic corticosteroids	35,421 (57.86)	23,697 (61.90)	− 0.082	16,055 (59.33)	16,069 (59.38)	− 0.001
Antibiotics	46,719 (76.31)	29,552 (77.19)	− 0.021	20,450 (75.57)	20,442 (75.54)	0.001
Healthcare services, mean (SD)						
Pneumococcal or influenza vaccination	25,457 (41.58)	15,797 (41.26)	0.007	11,040 (40.80)	11,122 (41.10)	− 0.006
No. of any outpatient visit	39.66 (26.04)	40.05 (26.41)	− 0.015	39.32 (26.41)	39.29 (26.24)	0.001
No. of outpatient visit due to COPD	5.23 (6.54)	4.40 (5.83)	0.134	4.34 (5.77)	4.43 (5.81)	− 0.016
No. of outpatient visit due to cardiovascular diseases^c^	11.13 (12.40)	11.34 (12.76)	− 0.017	10.91 (12.30)	10.99 (12.45)	− 0.006
No. of any hospitalization	0.82 (1.56)	0.73 (1.55)	0.052	0.65 (1.32)	0.66 (1.48)	− 0.008
No. of hospitalization due to COPD	0.28 (0.75)	0.23 (0.69)	0.077	0.18 (0.58)	0.19 (0.65)	− 0.009
No. of hospitalizations due to cardiovascular disease^c^	0.52 (1.12)	0.48 (1.11)	0.035	0.42 (0.97)	0.43 (1.06)	− 0.007

*ACEI* angiotensin converting enzyme inhibitors, *ARB* angiotensin receptor blockers, *COPD* chronic obstructive pulmonary disease, *FDC* fixed-dose combinations, *ICS* inhaled corticosteroids, *LABA* long-acting β_2_ agonists, *LAMA* long-acting muscarinic antagonists, *NSAID* non-steroidal anti-inflammatory drugs, *PS* propensity score, *SD* standard deviation

^a^One randomly sampled LABA/LAMA FDC initiator versus one LABA/ICS FDC initiator in each matched subset

^b^COPD duration was defined as the duration from the first recorded date of COPD diagnosis (looking back until 2014/01/01) to the cohort entry date

^c^Cardiovascular disease include hypertension, ischemic heart disease or angina, myocardial infarction, coronary revascularization, cardiac dysrhythmia, congestive heart failure, cerebrovascular disease, ischemic stroke, hemorrhagic stroke, transient ischemic attack, peripheral vascular disease, diabetes mellitus, and hyperlipidemia

### Risk of cardiovascular events associated with LABA/LAMA FDC compared to LABA/ICS FDC

#### Composite cardiovascular events

Before PS matching and for composite cardiovascular events, the mean follow-up duration was 256 days for LABA/LAMA FDC initiators and 163 days for LABA/ICS FDC initiators. The crude incidence rates for LABA/LAMA FDC and LABA/ICS FDC initiators were 24.97 and 30.74 per 1000 person-years, respectively (Table 2), corresponding to a crude HR of 0.92 (95% CI, 0.83–1.02) (Table 3). After PS matching, the incidence rates were 25.22 and 30.90 per 1000 person-years among LABA/LAMA FDC and LABA/ICS FDC initiators, respectively (Table 2). Use of LABA/LAMA FDC did not show a higher risk compared to LABA/ICS FDC (HR after PS matching, 0.89; 95% CI, 0.78–1.01). The cumulative incidence plots of the composite cardiovascular events were consistent with above findings (Additional file 1: Fig. S3).

**Table 2:** Number of patients and events, follow-up duration, and incidence rate of composite and individual cardiovascular events among LABA/LAMA FDC initiators and LABA/ICS FDC initiators before and after PS matching

	Before PS matching (n = 99,506)		After PS matching (n = 75,926)	
	LABA/LAMA FDC	LABA/ICS FDC	LABA/LAMA FDC	LABA/ICS FDC
	n = 61,221	n = 38,285	n = 48,864	n = 27,062
Composite cardiovascular events				
Number of events	1070	526	819	361
Mean follow-up days (SD)	255.68 (277.05)	163.22 (185.55)	247.36 (272.07)	157.69 (177.80)
Incidence rate (95% CI)^a^	24.97 (23.52–26.51)	30.74 (28.23–33.49)	25.22 (22.96–27.69)	30.90 (27.87–34.26)
Acute myocardial infarction				
Number of events	225	120	174	83
Mean follow-up days (SD)	257.91 (278.57)	164.26 (186.23)	249.46 (273.48)	158.66 (178.40)
Incidence rate (95% CI)^a^	5.20 (4.57–5.93)	6.97 (5.83–8.33)	5.28 (4.31–6.47)	7.06 (5.69–8.76)
Unstable angina				
Number of events	35	14	28	9
Mean follow-up days (SD)	258.39 (279.00)	164.52 (186.44)	249.84 (273.76)	158.94 (178.65)
Incidence rate (95% CI)^a^	0.81 (0.58–1.13)	0.81 (0.48–1.37)	0.97 (0.61–1.56)	0.76 (0.40–1.47)
Congestive heart failure				
Number of events	401	200	304	131
Mean follow-up days (SD)	257.45 (278.52)	164.01 (186.17)	248.98 (273.39)	158.49 (178.48)
Incidence rate (95% CI)^a^	9.29 (8.43–10.25)	11.63 (10.13–13.36)	9.42 (8.09–10.97)	11.16 (9.40–13.24)
Cardiac dysrhythmia				
Number of events	54	30	43	25
Mean follow-up days (SD)	258.41 (279.01)	164.52 (186.44)	249.86 (273.75)	158.93 (178.64)
Incidence rate (95% CI)^a^	1.25 (0.95–1.63)	1.74 (1.22–2.49)	1.34 (0.90–2.01)	2.12 (1.43–3.14)
Ischemic stroke				
Number of events	381	179	291	126
Mean follow-up days (SD)	257.42 (278.07)	164.09 (186.07)	248.89 (272.81)	158.50 (178.26)
Incidence rate (95% CI)^a^	8.83 (7.99–9.76)	10.41 (8.99–12.05)	8.62 (7.35–10.11)	10.73 (9.01–12.78)

*CI* confidence interval, *FDC* fixed-dose combinations, *ICS* inhaled corticosteroids, *LABA* long-acting β_2_ agonists, *LAMA* long-acting muscarinic antagonists, *PS* propensity score, *SD* standard deviation

^a^The unit of incidence rate was per 1000 person-years. The incidence rate after PS matching was weighted by the inverse of the matching ratio

**Table 3:** Risk of composite and individual cardiovascular events comparing LABA/LAMA FDC versus LABA/ICS FDC before and after PS matching

	Crude HR (95% CI)	HR after PS matching (95% CI)^a^
Composite cardiovascular events	0.92 (0.83–1.02)	0.89 (0.78–1.01)
Individual cardiovascular events		
Acute myocardial infarction	0.84 (0.67–1.06)	0.80 (0.61–1.05)
Unstable angina	1.80 (0.64–2.24)	1.48 (0.68–3.25)
Congestive heart failure	0.98 (0.83–1.17)	1.00 (0.80–1.24)
Cardiac dysrhythmia	0.75 (0.48–1.18)	0.62 (0.37–1.05)
Ischemic stroke	0.88 (0.74–1.06)	0.82 (0.66–1.02)

*CI* confidence interval, *FDC* fixed-dose combinations, *HR* hazards ratio, *ICS* inhaled corticosteroids, *LABA* long-acting β_2_ agonists, *LAMA* long-acting muscarinic antagonists, *PS* propensity score

^a^The HR after PS matching was stratified on the matching ratio

#### Individual cardiovascular events

The majority of cardiovascular events were congestive heart failure, followed by ischemic stroke and acute myocardial infarction (Table 2). Use of LABA/LAMA FDC was not observed with an evident, increased risk of individual outcomes compared to LABA/ICS FDC although there was a numerically elevated risk for unstable angina. The HRs after PS matching were 0.80 (95% CI, 0.61–1.05) for acute myocardial infarction, 1.48 (95% CI, 0.68–3.25) for unstable angina, 1.00 (95% CI, 0.80–1.24) for congestive heart failure, 0.62 (95% CI, 0.37–1.05) for cardiac arrhythmia, and 0.82 (95% CI, 0.66–1.02) for ischemic stroke (Table 3).

### Findings of the positive control outcome

LABA/LAMA FDC initiators had a lower rate of pneumonia compared to LABA/ICS FDC initiators during follow-up, corresponding to an HR after PS matching of 0.65 (95% CI, 0.58–0.74) (Additional file 1: Table S10).

### Findings of sensitivity analyses

The intention-to-treat approach yielded mean follow-up days of 651 days for LABA/LAMA FDC initiators and 676 days for LABA/ICS FDC initiators, corresponding to an HR after PS matching of 0.97 (95% CI, 0.90-1.05) (Table 4; Additional file 1: Table S11). After accounting for competing risk from overall death, the cumulative incidence plots tended to be slightly flatter (Additional file 1: Fig. S4) compared to that of the main analysis. However, the Fine-Gray subdistribution HR after PS matching (0.90 [95% CI, 0.79–1.03], see Table 4) did not change materially when compared to what was generated by the traditional Cox model. The hd-PS estimation also yielded a similar finding (HRs after PS matching, 0.88 [95% CI, 0.77-1.00], see Table 4).

**Table 4:** Sensitivity analyses for risk of composite cardiovascular events comparing LABA/LAMA FDC versus LABA/ICS FDC after PS matching

	LABA/LAMA FDC	LABA/ICS FDC	HR after PS matching (95% CI)^a^
	Events/patients		
Intention-to-treat approach	1852/48,864	1041/27,062	0.97 (0.90–1.05)
Fine-Gray approach	819/48,864	361/27,062	0.90 (0.79–1.03)
Including additional empirical claims-based covariates identified by hd-PS estimation in the PS model	832/48,842	364/27,027	0.88 (0.77–1.00)
Incorporating additional clinical parameters in the PS model			
- Laboratory test only	819/48,853	370/26,893	0.73 (0.31–1.73)
- Laboratory test, lung function test, respiratory symptoms, blood pressure, and health behavior	833/48,640	361/26,326	0.89 (0.77–1.03)

*CI* confidence interval, *FDC* fixed-dose combinations, *hd* high-dimensional, *HR* hazards ratio, *ICS* inhaled corticosteroids, *LABA* long-acting β_2_ agonists, *LAMA* long-acting muscarinic antagonists, *PS* propensity score

^a^The HR after PS matching was stratified on the inverse of the matching ratio

A total of 83,025 patients (83% of the eligible patients) had at least one clinical parameter captured from either the NHI Laboratory Database or the COPD P4P Database Information from the NHI Laboratory Database tended to be more comprehensive (approximately 82% of patients having at least one laboratory test result). Information from the COPD P4P Database; however, was less complete (approximately 8% of patients having data of lung function test, respiratory symptoms, blood pressure, or health behavior) (Additional file 1: Table S12).When we applied multiple imputation to incorporate these additional parameters in the PS-matched analysis, the risk estimate did not change apparently from those in the main analysis (HRs of 0.73 [95% CI, 0.31–1.73] when only including data from the NHI Laboratory Database and 0.89 [95% CI, 0.77–1.03] when simultaneously including data from the NHI Laboratory Database and the COPD P4P Database) (Table 4). Distribution of these parameters between FDC treatment groups became balanced as well (Additional file 1: Table S13).

### Findings of subgroup analyses

**Fig. 2:**
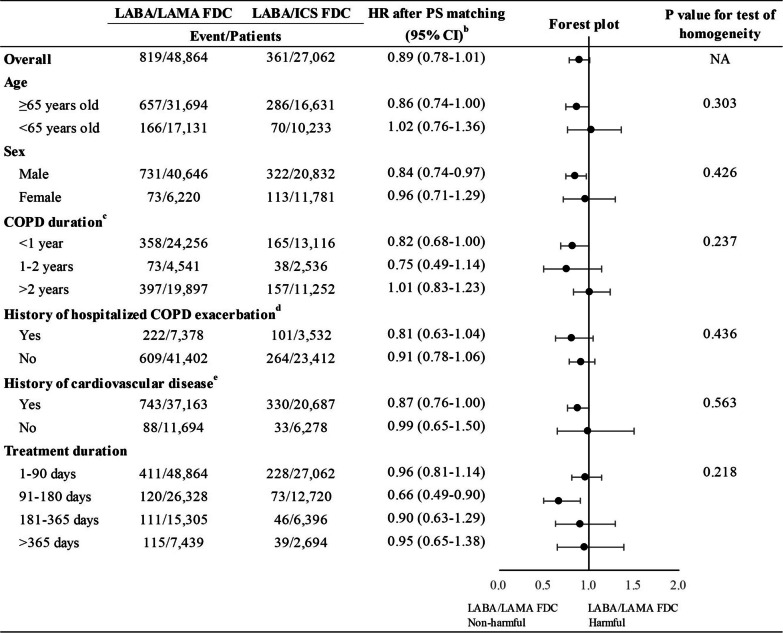
Subgroup analyses for risk of composite cardiovascular events comparing LABA/LAMA FDC with LABA/ICS FDC after PS matching, by patient characteritstic and treatment duration^a^. *CI* confidence interval, *COPD* chronic obstructive pulmonary disease, *FDC* fixed-dose combinations, *HR* hazards ratio, *ICS* inhaled corticosteroids, *LABA* long-acting β_2_ agonists, *LAMA* long-acting muscarinic antagonists, *NA* not applicable, *PS* propensity score. ^a^We re-estimated PS and re-matched patients in each patient subgroup. ^b^The HR after PS matching was stratified on the matching ratio. ^c^COPD duration was defined as the duration from the first recorded date of COPD diagnosis (looking back until 2014/01/01) to the cohort entry date. ^d^History of hospitalized COPD exacerbations was defined as having hospitalized COPD exacerbations within 365 days before cohort entry based on any diagnosis positions in the inpatient claims. ^e^History of cardiovascular diseases was defined as having the following cardiovascular diseases within 365 days before cohort entry based on any diagnosis or procedure positions or health services records in the outpatient and inpatient claims, including hypertension, ischemic heart disease or angina, myocardial infarction, coronary revascularization, cardiac dysrhythmia, congestive heart failure, cerebrovascular disease, ischemic stroke, hemorrhagic strike, peripheral vascular disease, diabetes, and hyperlipidemia

Overall, the risk of composite cardiovascular events associated with LABA/LAMA FDC did not change materially by patient characteristic (Figure 2; Additional file 1: Table S14), individual LABA/LAMA FDC and LABA/ICS FDC (Additional file 1: Table S15), and treatment duration (Figure 2; Additional file 1: Table S16), although the risk seemed to be lower among some subgroups. The p-values for test of homogeneity across patient subgroups were > 0.05.

## Discussion

This nationwide cohort study with >75,000 eligible patients did not observe an increased cardiovascular risk associated with LABA/LAMA FDC compared to LABA/ICS FDC. The sensitivity analyses with a longer follow-up duration, accounting for competing risk from overall death, and addressing potential unmeasured confounding yielded similar findings. The subgroup analyses, stratified by patient characteristic, comparing individual LABA/LAMA FDC to LABA/ICS FDC, and examining different treatment duration, also did not show evident variations in risk estimates across each patient subgroups. Our results provide important reassurance about the comparative safety of dual bronchodilator treatment strategies in COPD in the real-world settings.

### Comparison with existing clinical trials

One meta-analysis of clinical trials (n = 18,170) reported an increased risk of MACE comparing LABA/LAMA to LABA/ICS (1.6% vs 1.3%; risk ratio, 1.42 [95% CI, 1.11–1.81]) [6]. The results were mainly driven by one dual combination therapy trial (LABA/LAMA, LABA/ICS) and two triple combination therapy trials (LABA/LAMA/ICS, LABA/LAMA, LABA/ICS) (n = 13,817; 2.0% vs 1.5%; risk ratio, 1.40 [95% CI, 1.08–1.82]) [9–11]. The discrepant findings generated from these trial data and our study should be elaborated carefully in terms of differences in patient characteristics and methodological concerns across data source.

Specifically, given the inclusion and exclusion criteria, patients enrolled in these efficacy trials tended to have more severe COPD disease but had no clinically significant cardiovascular abnormalities. As demonstrated in Additional file 1: Table S17, patients in these trials had mean COPD duration of 7–8 years and mean predicted FEV_1_ of 44–56%; all the patients had moderate or severe exacerbation history (> 20% having at least one severe exacerbation episode); the mean CAT score was 17–20; and nearly 56–81% of enrollees had baseline ICS use and some may have prior asthma history. In terms of methodological perspectives, the trial design of forced ICS withdrawal at randomization may therefore exacerbate disease control for those who have benefited from ICS treatment but were allocated to the LABA/LAMA group [12, 13], potentially leading to subsequent cardiovascular consequences. Moreover, the primary endpoint of the three trials was an annual rate of COPD exacerbations rather than cardiovascular outcomes. This may raise concerns about adjudication and potential misclassification of cardiovascular events in the original trials and in the meta-analysis [54]. This may also result in lack of detailed data on time to cardiovascular events for HR estimation.

The present Taiwanese cohort study included patients with COPD who initiated LABA/LAMA FDC or LABA/ICS FDC in the daily practice, who tended to have mild COPD disease but may have major underlying cardiovascular disease compared to enrollees in aforementioned trials. For example, our study patients had mean COPD duration of 2 years (measured in the database) and mean predicted FEV_1_ of 63% (mainly based on imputed data); 14% of patients had at least one severe exacerbation episode; the mean CAT score was 13 (mainly based on imputed data); and only 10% of patients ever used ICS at baseline (Additional file 1: Table S17). To mitigate misclassification of cardiovascular outcomes, we ascertained cardiovascular events according to validated algorithms. Our on-treatment and intention-to-treat follow-up approaches yielded similar results, which did not show a higher cardiovascular risk associated with LABA/LAMA FDC use. Our findings highlight that it deserves more caution when directly applying trial data for safety evaluation in the real-world settings.

### Comparison with existing real-world studies

Few real-world studies have evaluated cardiovascular risk of LABA/LAMA versus LABA/ICS. One cohort study using the US Truven Health MarketScan Commercial and Medicare Database (2004–2012, n = 19,078) did not observe an increased risk of hospitalized, composite cardiovascular events (HR, 0.85; 95% CI, 0.66–1.04) [7]. Use of LABA/LAMA or LABA/ICS in this study; however, included FDC and free-combination forms. Another cohort study conducted in the Taiwan NHIRD (2015–2016, n = 28,237) further showed that there was no excess risk of hospitalized, composite cardiovascular events between individual LABA/LAMA FDC and LABA/ICS FDC. The HRs ranged from 1.03 (95% CI, 0.83–1.29) to 1.29 (95% CI, 0.96–1.73) comparing two LABA/LAMA FDC (IND/GLY, VIL/UME) to three LABA/ICS FDC (FOR/BEC, FOR/BUD, SAL/FLU) [8].

Using the Taiwan NHIRD with more updated data (2017–2020, n = 75,926), the present cohort study yielded a larger sample size and included more FDC products (three LABA/LAMA FDC [IND/GLY, VIL/UME, OLO/TIO] and five LABA/ICS FDC [FOR/BEC, FOR/BUD, FOR/FLU, SAL/FLU, VIL/FLU]). Extending findings from prior cohort studies, we comprehensively presented the risk of various cardiovascular outcomes and the risk across patients with different characteristics (such as patients with hospitalized COPD exacerbations or with cardiovascular disease at baseline), with different comparisons of LABA/LAMA FDC versus LABA/ICS FDC, and with longer treatment durations (such as > 365 days). The results provide more informative messages and facilitate physicians for treatment decision in different clinical scenarios.

### Strengths and limitation of the present study

Our study has some notable strengths. First, to our knowledge, our study is the largest study which provides real-world evidence on cardiovascular safety of LABA/LAMA FDC versus LABA/ICS FDC. Second, under a target trial emulation framework, we defined study population, treatment initiation, outcome occurrence, and baseline covariates anchored at the cohort entry date (i.e., T_0_). This prevented a common shortcoming of “looking forward the future” and accompanying bias in the real-world studies [18–22]. Third, in alignment with the emulation framework, our main analysis extracted diagnosis and medication information from the Taiwan NHIRD derived from a single-payer health insurance system. We used validated claim-based algorithms to determine cardiovascular and pneumonia outcomes. We also replicated the known association between LABA/ICS FDC and pneumonia. To mitigate potential confounding by patient characteristic, we applied the state-of-the-art PS matching analysis accounting for > 80 pre-specified claims-based covariates that may be associated with the exposures and the outcomes. Collectively, these lend support to our study validity. Fourth, we further improved confounding control using the sophisticated hd-PS algorithm with additional empirical claims-based covariates and the multiple imputation approach with additional important clinical parameters. Fifth, the findings of several subgroup analyses enhance the generalizability and the application of our study.

We need to recognize some limitations. First, our study yielded a mean follow-up duration of 215 days (based on an on-treatment approach), which tended to be shorter than that in previous clinical trials (52-week follow-up duration based on an intention-to-treat approach) [9–11]. However, this reflected actual use patterns of LABA/LAMA FDC in the real-world environment and was consistent with previous observational studies [7, 8]. Our sensitivity analysis based on an intention-to-treat approach (a mean follow-up duration of 660 days, Table 4 and Additional file 1: Table S11) and subgroup analysis with longer treatment duration (such as >365 days, Figure 2 and Additional file 1: Table S16) did not change the results materially. Second, we examine a broad spectrum of cardiovascular events to enhance clinical relevance. However, the current database did not provide information on cardiovascular death for further risk estimation. Third, while we controlled for a large number of potential confounders with several approaches, we could not fully exclude the possibility of residual or unmeasured confounding, which was an inherent limitation of most observational studies. Similarly, we attempted to integrate claims-based covariates and clinical parameters to strengthen confounding adjustment. For example, for adjusting for the influence of COPD severity, we simultaneously captured information on COPD duration, history of hospitalized COPD exacerbations, and lung function test results. However, proportions of patients with clinical parameters from COPD P4P Database tended to be low. This may be because the database was only available since 2017. The availability with corresponding information is expected to increase over time and future studies with accrual data would facilitate replication of our results.

## Conclusion

In this nationwide cohort study conducted with a target trial emulation framework, there was no substantial increased risk associated with LABA/LAMA FDC compared to LABA/ICS FDC. The concerns of sympathetic activation and cardiovascular events may not preclude dual bronchodilator treatment for patients with appropriate indication.

### Take home message

This population-based cohort study with a target trial emulation framework provides important reassurance about comparative cardiovascular safety of LABA/LAMA FDC compared with LABA/ICS FDC among patients with COPD.

### Supplementary Information

**Additional file 1: Table S1**. Specification and emulation of a target trial of LABA/LAMA FDC versus LABAICS FDC among patients with COPD using real-world data from Taiwan NHIRD. **Table S2**. International Classification of Diseases, 9th or 10th Revision, Clinical Modification diagnosis codes used to identify patients with COPD. **Table S3**. Anatomical Therapeutic Chemical classification system codes used to identify use of LABA/LAMA FDC or LABA/ICS FDC. **Table S4**. International Classification of Diseases, 9th or 10th Revision, Clinical Modification diagnosis codes used to identify outcomes of interest and the positive control outcome. **Table S5**. International Classification of Diseases, 9th or 10th Revision, Clinical Modification diagnosis or procedure codes or Taiwan health insurance service claims codes used to identify comorbidities and measures of healthcare services at baseline. **Table S6**. Anatomical Therapeutic Chemical classification system codes used to identify medication use at baseline. **Table S7**. Measurement of the 11 clinical parameters at baseline. **Table S8**. Summary of subgroup analyses. **Table S9**. Patient characteristics of the eligible cohort before and after PS matching. **Table S10**. Number of patients and events, follow-up duration, incidence rate, and risk of pneumonia comparing LABA/LAMA FDC with LABA/ICS FDC before and after PS matching. **Table S11**. Number of patients and events, follow-up duration, incidence rate, and risk of composite cardiovascular events comparing LABA/LAMA FDC with LABA/ICS FDC before and after PS matching, by intention-to-treat approach. **Table S12**. Availability of the clinical parameters at baseline in the eligible cohort. **Table S13**. Clinical parameters of the imputed cohort before and after PS matching. **Table S14**. Number of patients, number of events, and risk of composite cardiovascular events comparing LABA/LAMA FDC versus LABA/ICS FDC before and after PS matching, by patient characteristic. **Table S15**. Number of patients, number of events, and risk of composite cardiovascular events comparing LABA/LAMA FDC versus LABA/ICS FDC before and after PS score matching, by individual LABA/LAMA FDC and LABA/ICS FDC. **Table S16**. Number of patients, number of events, and risk of composite cardiovascular events comparing LABA/LAMA FDC versus LABA/ICS FDC before and after PS matching, by treatment duration. **Table S17**. Selected patient characteristics at baseline and cardiovascular outcomes during follow-up of three substantial efficacy trials and our study. **Figure S1**. Study cohort assembly. **Figure S2**. Distributions of propensity score by study drug before and after PS matching. **Figure S3**. Cumulative incidence plots of composite cardiovascular events by study FDC treatment before and after PS matching derived from complement of the Kaplan-Meier survival function. **Figure S4**. Cumulative incidence plots of composite cardiovascular events by study FDC treatment before and after PS matching accounting for the influence of competing risk from overall death.

## Data Availability

The data used in the current study were obtained from the Applied Health Research Data Integration Service from the National Health Insurance Administration, Taiwan, which are not publicly available given the data protection policy. However, the authors are willing to have further discussion if there are any questions.
