# Supplementary material for: Comparative cardiovascular safety of LABA/LAMA FDC versus LABA/ICS FDC in patients with chronic obstructive pulmonary disease: a population-based cohort study with a target trial emulation framework
Source: Respir Res. 2023 Sep 29;24:239. doi: 10.1186/s12931-023-02545-9 (PMC10543303; doi:10.1186/s12931-023-02545-9)
Supplement: Supplementary file 1 — Additional file 1: Table S1. Specification and emulation of a target trial of LABA/LAMA FDC versus LABAICS FDC among patients with COPD using real-world data from Taiwan NHIRD. Table S2. International Classification of Diseases, 9th or 10th Revision, Clinical Modification diagnosis codes used to identify patients with COPD. Table S3. Anatomical Therapeutic Chemical classification system codes used to identify use of LABA/LAMA FDC or LABA/ICS FDC. Table S4. International Classification of Diseases, 9th or 10th Revision, Clinical Modification diagnosis codes used to identify outcomes of interest and the positive control outcome. Table S5. International Classification of Diseases, 9th or 10th Revision, Clinical Modification diagnosis or procedure codes or Taiwan health insurance service claims codes used to identify comorbidities and measures of healthcare services at baseline. Table S6. Anatomical Therapeutic Chemical classification system codes used to identify medication use at baseline. Table S7. Measurement of the 11 clinical parameters at baseline. Table S8. Summary of subgroup analyses. Table S9. Patient characteristics of the eligible cohort before and after PS matching. Table S10. Number of patients and events, follow-up duration, incidence rate, and risk of pneumonia comparing LABA/LAMA FDC with LABA/ICS FDC before and after PS matching. Table S11. Number of patients and events, follow-up duration, incidence rate, and risk of composite cardiovascular events comparing LABA/LAMA FDC with LABA/ICS FDC before and after PS matching, by intention-to-treat approach. Table S12. Availability of the clinical parameters at baseline in the eligible cohort. Table S13. Clinical parameters of the imputed cohort before and after PS matching. Table S14. Number of patients, number of events, and risk of composite cardiovascular events comparing LABA/LAMA FDC versus LABA/ICS FDC before and after PS matching, by patient characteristic. Table S15. Number of patients, number of ev [file 12931_2023_2545_MOESM1_ESM.docx]

**Comparative cardiovascular safety of LABA/LAMA FDC versus LABA/ICS FDC in patients with chronic obstructive pulmonary disease: a population-based cohort study with a target trial emulation framework**

| **eMethods** |  |
| --- | --- |
| Data source | 4 |
|  |  |
| Exclusion criteria | 5 |
|  |  |
| Application of variable-ratio matching for confounding control | 5 |
|  |  |
| Application of the Fine‐Gray subdistribution hazard model to account for the influence of competing risk from overall death | 6 |
|  |  |
| Application of high-dimensional PS estimation to incorporate 100 additional empirically-identified, claims-based covariates into the PS model | 6 |
|  |  |
| Application of multiple imputation to incorporate the 11 additional clinical parameters into the PS model | 6 |
|  |  |
| **Tables** |  |
| Table S1. Specification and emulation of a target trial of LABA/LAMA FDC versus LABAICS FDC among patients with COPD using real-world data from Taiwan NHIRD | 8 |
|  |  |
| Table S2. International Classification of Diseases, 9^th^ or 10^th^ Revision, Clinical Modification diagnosis codes used to identify patients with COPD | 11 |
|  |  |
| Table S3. Anatomical Therapeutic Chemical classification system codes used to identify use of LABA/LAMA FDC or LABA/ICS FDC | 12 |
|  |  |
| Table S4. International Classification of Diseases, 9^th^ or 10^th^ Revision, Clinical Modification diagnosis codes used to identify outcomes of interest and the positive control outcome | 13 |
|  |  |
| Table S5. International Classification of Diseases, 9^th^ or 10^th^ Revision, Clinical Modification diagnosis or procedure codes or Taiwan health insurance service claims codes used to identify comorbidities and measures of healthcare services at baseline | 14 |
|  |  |
| Table S6. Anatomical Therapeutic Chemical classification system codes used to identify medication use at baseline | 18 |
|  |  |
| Table S7. Measurement of the 11 clinical parameters at baseline | 20 |
|  |  |
| Table S8. Summary of subgroup analyses | 21 |
|  |  |
| Table S9. Patient characteristics of the eligible cohort before and after PS matching | 23 |
|  |  |
| Table S10. Number of patients and events, follow-up duration, incidence rate, and risk of pneumonia comparing LABA/LAMA FDC with LABA/ICS FDC before and after PS matching | 28 |
|  |  |
| Table S11. Number of patients and events, follow-up duration, incidence rate, and risk of composite cardiovascular events comparing LABA/LAMA FDC with LABA/ICS FDC before and after PS matching, by intention-to-treat approach | 29 |
|  |  |
| Table S12. Availability of the clinical parameters at baseline in the eligible cohort | 30 |
|  |  |
| Table S13. Clinical parameters of the imputed cohort before and after PS matching | 31 |
|  |  |
| Table S14. Number of patients, number of events, and risk of composite cardiovascular events comparing LABA/LAMA FDC versus LABA/ICS FDC before and after PS matching, by patient characteristic | 33 |
|  |  |
| Table S15. Number of patients, number of events, and risk of composite cardiovascular events comparing LABA/LAMA FDC versus LABA/ICS FDC before and after PS score matching, by individual LABA/LAMA FDC and LABA/ICS FDC | 35 |
|  |  |
| Table S16. Number of patients, number of events, and risk of composite cardiovascular events comparing LABA/LAMA FDC versus LABA/ICS FDC before and after PS matching, by treatment duration | 37 |
|  |  |
| Table S17. Selected patient characteristics at baseline and cardiovascular outcomes during follow-up of three substantial efficacy trials and our study | 38 |
|  |  |
| **Figures** |  |
| Figure S1. Study cohort assembly | 41 |
|  |  |
| Figure S2. Distributions of propensity score by study drug before and after PS matching | 42 |
|  |  |
| Figure S3. Cumulative incidence plots of composite cardiovascular events by study FDC treatment before and after PS matching derived from complement of the Kaplan-Meier survival function | 43 |
|  |  |
| Figure S4. Cumulative incidence plots of composite cardiovascular events by study FDC treatment before and after PS matching accounting for the influence of competing risk from overall death | 44 |
|  |  |
| **eReferences** | 45 |

**eMethods**

**Data source**

The present study used three nationwide databases established under series of national health policies of the National Health Insurance Administration (NHIA) in Taiwan, including the Taiwan National Health Insurance Database (NHIRD), the Taiwan National Health Insurance (NHI) Laboratory Database, and the Taiwan chronic obstructive pulmonary disease pay-for-performance (COPD P4P) Database. Specifically, we identified study population and ascertained information on exposure, outcomes, and covariates for the main analysis using claims data in the NHIRD. To enhance confounding control, we additionally captured several clinical parameters from the NHI Laboratory Database and the COPD P4P Database for the sensitivity analysis. See below for detailed data source description.

Taiwan NHIRD

In 1995, the NHIA initiated a single-payer, compulsory national health insurance (NHI) program in Taiwan, which enrolled 99% of the 23-million residents by June of 2021.[1] The Taiwan NHIRD, which was established based on claims data generated from the NHI program and is maintained periodically by the NHIA, thereby includes comprehensive demographic and enrollment information, diagnosis and procedure records, and pharmacy dispensing claims from outpatient visits and hospital admissions.[2]

Taiwan NHI Laboratory Database

To enhance disease care, prevent wasting medical resource (e.g., duplication of laboratory test orders), and enrich data dimensions of the NHIRD, since 2015, the NHIA has encouraged healthcare facilities to upload laboratory test results of each encounter to the NHIA and built up the NHI Laboratory Database. There were more than 600 types of laboratory tests available by January of 2020 and the most commonly performed tests were whole blood count, urine routine, white blood cell differential count, blood sugar, and blood creatinine.[3]

Taiwan COPD P4P Database

To improve the quality of pharmacological and non-pharmacological treatment and reduce disease progression among patients with COPD, since 2017, the NHIA has launched a nationwide COPD P4P program. The COPD P4P Database has therefore been available, which contains information on important clinical parameters for patients enrolled in the program, including results of lung function test, respiratory symptoms, blood pressure, and health behavior.[4]

These three databases were obtained from the Applied Health Research Data Integration Service from the NHIA, Taiwan. All data are de-identified but linkable across databases. The National Yang-Ming Chiao Tung University Research Ethics Committee approved the study. Informed consent was waived given the retrospective nature of the study and the analysis of anonymous data.

**Exclusion criteria**

Our study population comprised patients with COPD who initiated LABA/LAMA FDC or LABA/ICS FDC from the NHIRD between 2017/01/01 and 2020/12/31. The cohort entry date (i.e., T0) was the date of the first dispensing of a LABA/LAMA FDC or LABA/ICS FDC after a diagnosis of COPD.

We excluded patients aged <40 years or >100 years at COPD diagnosis and those with ambiguous or missing age or sex information. To describe patient characteristics more completely, we required patients to have interactions with the healthcare systems by excluding those without any outpatient or inpatient visits within 365 days before cohort entry. To implement a new-user design, we required patients who initiated LABA/LAMA FDC or LABA/ICS FDC treatment to have no dispensing of any study FDC drugs within the same 365-day window. We also excluded patients who received both LABA/LAMA FDC and LABA/ICS FDC; patients who simultaneously received LABA, LAMA, and ICS; or patients who simultaneously received more than one LABA/LAMA FDC or one LABA/ICS FDC at cohort entry. Given that LABA/LAMA FDC is not indicated for asthma, we excluded patients with an asthma diagnosis within 365 days before and at cohort entry. As part of the data quality assurance process, we excluded a very small proportion of patients who had death records before cohort entry. Death was defined as disenrollment due to death or death during hospitalization. We also excluded a very small proportion of patients whose cohort entry date was 2020/12/31, i.e., the last date of the assessment period, given no opportunity to observe their outcome occurrence.

**Application of variable-ratio matching for confounding control**

The present study estimated the baseline propensity scores (PS), i.e., the probabilities of initiating LABA/LAMA FDC versus LABA/ICS FDC, with a logistic regression model including a comprehensive list of predefined, claims-based covariates. Because there were many more LABA/LAMA FDC initiators, up to five LABA/LAMA FDC initiators were matched to one LABA/ICS FDC initiator using a nearest-neighbor algorithm without replacement and with a maximum matching caliper of 0.025 on the PS scale.[5]

Variable-ratio matching produces covariate balance within matched sets but not marginally in the overall matched population. Therefore, all the analyses after PS matching should account for different matching ratios (1 to 5). Specifically, we (1) randomly selected one LABA/LAMA FDC initiator from each set of patients matched to each LABA/ICS FDC initiator and assessed whether adequate balance in covariates was achieved between FDC treatment groups using standardized differences[6] among this sample; (2) weighted the data with the inverse of the matching ratio to estimate incidence rates and generate cumulative incidence plots in the variable-ratio matched cohort; and (3) used Cox proportional hazards models stratified on the matching ratio to estimate hazards ratios (HRs) and 95% confidence intervals (CIs) after variable-ratio matching.

**Application of the Fine‐Gray subdistribution hazard model to account for the influence of competing risk from overall death**

In the real-world settings, a competing risk is an outcome whose occurrence prevents the occurrence of the outcome of interest. For example, patients with COPD usually have higher overall death (i.e., competing risk), which may considerably preclude observation of composite cardiovascular events (outcome of interest) during follow-up. To account for the influence of competing risk from overall death, we applied the Fine‐Gray subdistribution hazard model, which remains patients who have previously experienced overall death in the risk sets for subdistribution HR estimation. The subdistribution HR therefore can be interpreted as an instantaneous rate ratio of composite cardiovascular events comparing LABA/LAMA FDC versus LABA/ICS FDC among patients who have not yet experienced the events or patients who have previously died.[7,8]

**Application of high-dimensional PS (hd-PS) estimation to incorporate 100 additional empirically-identified, claims-based covariates into the PS model**

The hd-PS estimation is a semi-automated algorithm that identifies, prioritizes, and selects a large number of empirical, claims-based covariates that are simultaneously associated with exposure and the outcome for inclusion in the PS model. Specifically, we (1) identified the 200 most prevalent covariates within individual data dimensions, respectively, including outpatient and inpatient diagnoses, procedures, and medication use; (2) estimated the association between exposure (i.e. use of LABA/LAMA FDC) and the outcome (i.e., composite cardiovascular events) for each covariate separately; and (3) prioritized and selected top 100 covariates based on the magnitude of the association. These covariates therefore serve as proxies of confounders that are not predefined or that are imbalanced by chance between exposure and the outcome.[9,10]

We combined these empirically-identified, claims-based covariates with the predefined covariates to estimate baseline hd-PS for each patient by logistic regression. We applied hd-PS matching to mitigate potential unmeasured confounding.

**Application of multiple imputation to incorporate the 11 additional clinical parameters into the PS model**

We extracted 11 important clinical parameters at baseline, including laboratory test results (eosinophil, c-reactive protein, low-density lipoprotein-cholesterol, glycated hemoglobin, and glomerular filtration rate [GFR] or estimated GFR), lung function test results (predicted post-dose forced expiratory volume in one second [FEV_1_], post-dose FEV_1_/forced vital capacity), COPD Assessment Test score, systolic blood pressure, and health behavior (body mass index and smoking status), to enhance confounding control. Specifically, we included these clinical parameters, in addition to pre-specified claims-based covariates, into the PS model, underwent PS matching, and examined if the results varied materially. Because not all the patients had information on these parameters, we conducted multiple imputation to handle missing data issues with the following three steps: (1) imputation, (2) analysis (i.e., PS estimation and matching), and (3) pooling of HRs.[11-13]

Imputation

First, in the total eligible population, we built an imputation model with variables of 11 clinical parameters, >80 pre-specified claims-based covariates, exposure, outcome, and follow-up time. Assuming that the clinical parameters were missing at random and there was a joint distribution for all the variables, we applied the fully conditional specification method to impute 10 datasets with complete continuous or categorical data on the clinical parameters. This step was conducted using the “PROC MI” command in SAS.[12,13]

Analysis (i.e., PS estimation and matching)

Second, within each imputed dataset, we estimated PS with the clinical parameters and all the claims-based covariates, conducted 5:1variable-ratio PS matching, and estimated HRs and 95% CIs for the composite cardiovascular events after PS matching stratifying on the matching ratio.

Pooling of HRs

Finally, we used Rubin’s rules to summarize HRs and 95% CIs across imputed datasets. This step was conducted using the “PROC MIANALYZE” command in SAS.[12,13]

The methods applied in the present study were also described in our previous work.[14,15]

**Table S1.** Specification and emulation of a target trial of LABA/LAMA FDC versus LABAICS FDC among patients with COPD using real-world data from Taiwan NHIRD

| Component | Specification of a target trial | Emulation of a target trial using real-world data  (i.e., Cohort study using a target trial emulation framework) |
| --- | --- | --- |
| Aim | To examine comparative cardiovascular safety of LABA/LAMA FDC and LABA/ICS FDC | |
|  |  | |
| Principle | Ascertainment of study population, exposure, outcomes, and baseline covariates was anchored at T_0_  (see description below and **Figure 1** for the graphic depiction). | |
|  |  | |
| Eligibility | Inclusion criteria   1. A diagnosis of COPD between 2017/01/01 and 2020/12/31. 2. Initiation of LABA/LAMA FDC or LABA/ICS FDC.   Exclusion criteria   1. Aged ≤40 years or aged >100 years. 2. had an asthma diagnosis within 365 days before and at cohort entry. | Same as for the target trial (please refer to “Study population and exposure” in the main text).  To emulate the target trial which fit the real-world circumstances   1. We applied a validated algorithm to identify patients with a COPD diagnosis between 2017/01/01 and 2020/12/31. 2. We further exclude patients who 3. had ambiguous or missing information on age and sex. 4. did not have interactions with the healthcare system within 365 days before cohort entry. 5. received LABA/LAMA FDC or LABA/ICS FDC within 365 days before cohort entry. 6. simultaneously received LABA/LAMA FDC and LABA/ICS FDC at cohort entry. 7. simultaneously received LABA, LAMA, and ICS at cohort entry. 8. received more than one LABA/LAMA FDC or more than one LABA/ICS FDC at cohort entry. 9. had death records before cohort entry. 10. had a cohort entry date on 2020/12/31. |
| Treatment strategies | 1. Initiation of LABA/LAMA FDC or 2. Initiation of LABA/ICS FDC | Same as for the target trial (please refer to “Study population and exposure” in the main text). |
| Treatment assignment | Eligible patients were assigned to either treatment strategy by randomization.  T_0_ was the date when randomization was executed. | Similar to the target trial (please refer to “Study population and exposure”, “Covariates”, and “Statistical analyses” in the main text).  Eligible patients were assigned to either treatment strategy based on the first dispensing after COPD diagnosis between 2007/01/01 and 2020/12/31.  Randomization was emulated by matching the two study treatment group on PS (i.e., the probabilities of initiating LABA/LAMA FDC versus LABA/ICS FDC conditional on covariates measured at baseline) to control for confounding.  T_0_ was the date of the first dispensing of study treatment after COPD diagnosis, i.e., cohort entry date. |
| Outcomes | A composite of cardiovascular events was defined as the first hospitalization for acute myocardial infarction, unstable angina, congestive heart failure, cardiac dysrhythmia, or ischemic stroke after T_0._  Individual components of the composite outcome. | Same as for the target trial (please refer to “Study outcomes and follow-up” in the main text).  We applied validated algorithms to ascertained each outcome. |
| Follow-up | 1. On-treatment approach, which followed patients from cohort entry to outcome occurrence, study FDC treatment discontinuation or change, death, or end of the study, whichever came first. 2. Intention-to-treat approach, which followed patients from cohort entry to outcome occurrence, death, or end of the study, whichever came first. | Same as for the target trial (please refer to “Study outcomes and follow-up” and “Sensitivity analysis” in the main text). |
| Causal contrast | 1. On-treatment effect (main analysis) 2. Intention-to-treat effect (sensitivity analysis) | Same as for the target trial (please refer to “Study outcomes and follow-up” and “Statistical analyses” in the main text). |
| Statistical analyses | Cox proportional hazards model were used to estimate HRs and 95% CIs of composite and individual cardiovascular events comparing LABA/LAMA FDC with LABA/ICS FDC. | Same as for the target trial (please refer to “Statistical analysis” in the main text). |

CI, confidence interval; COPD, chronic obstructive pulmonary disease; FDC, fixed-dose combinations; HR, hazards ratio; ICS, inhaled corticosteroids; LABA, long-acting β_2_ agonists; LAMA, long-acting muscarinic antagonists; NHIRD, National Health Insurance Database; PS, propensity score.

**Table S2.** International Classification of Diseases, 9^th^ or 10^th^ Revision, Clinical Modification (ICD-9-CM or ICD-10-CM) diagnosis codes^a^ used to identify patients with COPD

| ICD-9-CM diagnosis codes | | ICD-10-CM diagnosis codes | |
| --- | --- | --- | --- |
| 491 | Chronic bronchitis | J41 | Simple and mucopurulent chronic bronchitis |
| 491.0 | Simple chronic bronchitis | J41.0 | Simple chronic bronchitis |
| 491.1 | Mucopurulent chronic bronchitis | J41.1 | Mucopurulent chronic bronchitis |
| 491.2 | Obstructive chronic bronchitis | J41.8 | Mixed simple and mucopurulent chronic bronchitis |
| 491.20 | Obstructive chronic bronchitis without exacerbation | J42 | Unspecified chronic bronchitis |
| 491.21 | Obstructive chronic bronchitis with (acute) exacerbation | J43 | Emphysema |
| 491.22 | Obstructive chronic bronchitis with acute bronchitis | J43.0 | MacLeod's syndrome |
| 491.8 | Other chronic bronchitis | J43.1 | Panlobular emphysema |
| 491.9 | Unspecified chronic bronchitis | J43.2 | Centrilobular emphysema |
| 492 | Emphysema | J43.8 | Other emphysema |
| 492.0 | Emphysematous bleb | J43.9 | Emphysema, unspecified |
| 492.8 | Other emphysema | J44 | Other chronic obstructive pulmonary disease |
| 496 | Chronic airway obstruction, not elsewhere classified | J44.0 | Chronic obstructive pulmonary disease with (acute) lower respiratory infection |
|  |  | J44.1 | Chronic obstructive pulmonary disease with (acute) exacerbation |
|  |  | J44.8 | Other specified chronic obstructive pulmonary disease |
|  |  | J44.9 | Chronic obstructive pulmonary disease, unspecified |

COPD, chronic obstructive pulmonary disease.

^a^Based on any diagnosis positions in the outpatient, inpatient, or emergency department claims.

**Table S3.** Anatomical Therapeutic Chemical (ATC) classification system codes used to identify use of LABA/LAMA FDC or LABA/ICS FDC^a,b^

| Medications (Brand name) | Abbreviation | ATC codes | Device | First reimbursement date | Reimbursement price (NTD) |
| --- | --- | --- | --- | --- | --- |
| LABA/LAMA FDC | | | | | |
| Vilanterol/Umeclidinium (Anoro^®^) | VIL/UME | R03AL03 | DPI | 2015/05/01 | 1,244-1,502 |
| Indacaterol/Glycopyrronium (Ultibro^®^) | IND/GLY | R03AL04 | DPI | 2015/01/01 | 1,221-1,611 |
| Olodaterol/Tiotropium (Spiolto^®^) | OLO/TIO | R03AL06 | SMI | 2016/11/01 | 1,530-1,585 |
| LABA/ICS FDC | | | | | |
| Salmeterol/Fluticasone (Seretide^®^, Airflusal^®^, Seroflo^®^) | SAL/FLU | R03AK06 | DPI, MDI | 2002/03/01 | 598-1,983 |
| Formoterol/Budesonide (Symbicort^®^) | FOR/BUD | R03AK07 | DPI, MDI | 2002/04/01 | 858-1,501 |
| Formoterol/Beclomethasone (Foster^®^) | FOR/BEC | R03AK08 | DPI, MDI | 2010/12/01 | 676-992 |
| Vilanterol/Fluticasone furoate (Relvar^®^) | VIL/FLU | R03AK10 | DPI | 2015/05/01 | 800-1,293 |
| Formoterol/Fluticasone (Flutiform^®^) | FOR/FLU | R03AK11 | MDI | 2015/06/01 | 598-1,190 |

DPI, dry powder inhaler; FDC, fixed-dose combination; ICS, Inhaled corticosteroids; LABA, long-acting β_2_ agonists; LAMA, long-acting muscarinic antagonists; MDI, metered dose inhaler; NTD, New Taiwan dollar; SMI, soft mist inhaler.

^a^Based on outpatient pharmacy dispensing claims.

^b^https://www.nhi.gov.tw/QueryN_New/QueryN/Query1 Accessed on April 21, 2023.

**Table S4.** International Classification of Diseases, 9^th^ or 10^th^ Revision, Clinical Modification (ICD-9-CM or ICD-10-CM) diagnosis codes^a^ used to identify outcomes of interest and the positive control outcome

| Outcomes | ICD-9-CM or ICD-10-CM diagnosis codes | PPV |
| --- | --- | --- |
| Outcome of interest^a^ | | |
| Acute myocardial infarction | ICD-9-CM diagnosis codes: 410  ICD-10-CM diagnosis codes: I21 | 92.9%[16]  100.0%[17] |
| Unstable angina | ICD-9-CM diagnosis codes: 411  ICD-10-CM diagnosis codes: I20.0 | 86%[18]  87.5%[19] |
| Congestive heart failure | ICD-9-CM diagnosis codes: 428  ICD-10-CM diagnosis codes: I50 | 93.7%[20]  95.9%[21] |
| Cardiac dysrhythmia | ICD-9-CM diagnosis codes: 427.1, 427.41, 427.42, 427.5, 798  ICD-10-CM diagnosis codes: I46.9, I47.2, I49.0, R99 | 79.7%[22,23]  NA |
| Ischemic stroke | ICD-9-CM diagnosis codes: 433, 434  ICD-10-CM diagnosis codes: I63 | 76.1%[24]  99.8%[25] |
| Positive control outcome^b^ | | |
| Pneumonia | I CD-9-CM diagnosis codes: 480-486  ICD-10-CM diagnosis codes: J12-J17, J18.0-J18.2, A481, A37.91, A22.1, B25.0, B44.0 | 88.0%[26]  NA |

PPV, positive predicted values; NA, not applicable.

^a^Based on the primary diagnosis positions in the inpatient claims.

^b^Based on any diagnosis positions in the inpatient claims.

**Table S5.** International Classification of Diseases, 9^th^ or 10^th^ Revision, Clinical Modification (ICD-9-CM or ICD-10-CM) diagnosis or procedure codes^a^ or Taiwan health insurance service claims codes^b^ used to identify comorbidities and measures of healthcare services at baseline

| Comorbidities | ICD-9-CM or ICD-10-CM diagnosis or procedure codes or Taiwan health insurance services claims codes |
| --- | --- |
|  | Comorbidities |
| Hypertension | ICD-9-CM diagnosis codes: 401-405  ICD-10-CM diagnosis codes: I10-I15, N26.2 |
| Ischemic heart disease or angina | ICD-9-CM diagnosis codes: 411, 413, 414  ICD-10-CM diagnosis codes: I20, I24, I25 |
| Myocardial infarction | ICD-9-CM diagnosis codes: 410, 412  ICD-10-CM diagnosis codes: I21, I22, I25.2 |
| Coronary revascularization | Coronary artery bypass grafting  ICD-9-CM procedure codes: 36.1, 36.2  ICD-10-CM procedure codes: 0210093, 02100A3, 20100J3, 02100K3, 02100Z3, 0210493, 02104A3, 02104J3, 02104K3, 02104Z3, 021009W, 02100AW, 02100JW, 02100KW, 021049W, 02104AW, 02104JW, 02104KW, 021109W, 02110AW, 02110JW, 02110KW, 021149W, 02114AW, 02114JW, 02114KW, 021209W, 02120AW, 02120JW, 02120KW, 021249W, 02124AW, 02124JW, 02124KW, 021309W, 02130AW, 02130JW, 02130KW, 021349W, 02134AW, 02134JW, 02134KW, 0210098, 0210099, 021009C, 02100A8, 02100A9, 02100AC, 02100J8, 02100J9, 02100JC, 02100K8, 02100K9, 02100KC, 02100Z8, 02100Z9, 02100ZC, 0210498, 0210499, 021049C, 02104A8, 02104A9, 02104AC, 02104J8, 02104J9, 02104JC, 02104K8, 02104K9, 02104KC, 02104Z8, 02104Z9, 02104ZC, 0211098, 0211099, 021109C, 02110A8, 02110A9, 02110AC, 02110J8, 02110J9, 02110JC, 02110J8, 02110K9, 02110KC, 02110Z8, 02110Z9, 02110ZC, 0211498, 0211499, 021149C, 02114A8, 02114A9, 02114AC, 02114J8, 02114J9, 02114JC, 02114K8, 02114K9, 02114KC, 02114Z8, 02114Z9, 02114ZC, 0212098, 0212099, 021209C, 02120A8, 02120A9, 02120AC, 02120J8, 02120J9, 02120JC, 02120K8, 02120K9, 02120KC, 02120Z8, 02120Z9, 02120ZC, 0212498, 0212499, 021249C, 02124A8, 02124A9, 02124AC, 02124J8, 02124J9, 02124JC, 02124K8, 02124K9, 02124KC, 02124Z8, 02124Z9, 02124ZC, 0213098, 0213099, 021309C, 02130A8, 02130A9, 02130AC, 02130J8, 02130J9, 02130JC, 02130K8, 02130K9, 02130KC, 02130Z8, 02130Z9, 02130ZC, 0213498, 0213499, 021349C, 02134A8, 02134A9, 02134AC, 02134J8, 20134J9, 02134JC, 02134K8, 02134K9, 02134KC, 02134Z8, 02134Z9, 02134ZC, 021009F, 02100AF, 02100JF, 02100KF, 021100ZF, 021049F, 02104AF, 02104JF, 02104KF, 02104ZF, 021109F, 02110AF, 02110JAF, 02110KF, 02110ZF, 021149F, 02114AF, 02114JF, 02114KF, 02114ZF, 021209F, 02120AF, 02120JF, 02120KF, 02120ZF, 021249F, 02124AF, 02124JF, 02124KF, 02124ZF, 021309F, 02130AF, 02130JF, 02130KF, 02130ZF, 021349F, 02134AF, 02134JF, 02134KF, 02134ZF, 0211093, 02110A3, 02110J3, 02110K3, 02110Z3, 0211493, 02114A3, 02114J3, 02114K3, 02114Z3, 0212093, 02120A3, 02120J3, 02120K3, 02120Z3, 0212493, 02124A3, 02124J3, 02124K3, 02124Z3, 0213093, 02130A3, 02130J3, 02130K3, 02130Z3, 0213493, 02134A3, 02134J3, 02134K3, 02134Z3  021K0Z8, 021K0Z9, 021K0ZC, 021K0ZF, 021K0ZW, 021K4Z8, 021K4Z9, 021K4ZC, 021K4ZF, 021K4ZW, 021L09P, 021L09Q, 021L09R, 021L0AP, 021L0AQ, 021L0AR, 021L0JP, 021L0JQ, 021L0JR, 021L0KP, 021L0KQ, 021L0KR, 021L0Z8, 021L0Z9, 021L0ZC, 021L0ZF, 021L0ZP, 021L0ZQ, 021L0ZR, 021L49P, 021L49Q, 021L49R, 021L4AP, 021L4AQ, 021L4AR, 021L4JP, 021L4JQ, 021L4JR, 021L4KP, 021L4KQ, 021L4KR, 021L4Z8, 021L4Z9, 021L4ZC, 021L4ZF, 021L4ZP, 021L4ZQ, 021L4ZR  Taiwan health insurance service claims codes: 68023A, 68023B, 68024A,68024B, 68025A, 68025B, 68053B, 68054B, 68055B, 83064A1, 97901K, 97902A, 97903B, 97906K, 97907A, 97908B, 97911K, 97912A, 97913B, 97916K, 97917A, 97918B, N26002, N26003  Percutaneous transluminal coronary angioplasty  ICD-9-CM procedure codes: 00.66, 36.01, 36.02, 36.05, 36.06, 36.07, 36.09,  ICD-10-CM procedure codes: 02703ZZ, 02704ZZ, 3E07317, 02713ZZ, 02714ZZ, 02723ZZ, 02724ZZ, 02733ZZ, 02734ZZ, 02C03ZZ, 02C04ZZ, 02C13ZZ, 02C14ZZ, 02C23ZZ, 02C24ZZ, 02C33ZZ, 02C34ZZ  Taiwan health insurance service claims codes: 33076A, 33076B, 33077A, 33077B, 33078A, 33078B, 97511K, 97512A, 97513B, 97516K, 97517A, 97518B, 97521K, 97522A, 97523B |
| Cardiac dysrhythmia | ICD-9-CM diagnosis codes: 427  ICD-10-CM diagnosis codes: I46-I49, R00.1 |
| Congestive heart failure | ICD-9-CM diagnosis codes: 428, 398.91, 402.01, 402.11, 402.91, 404.01, 404.11, 404.91, 404.03, 404.13, 404.93  ICD-10-CM diagnosis codes: I50, I09.81, I11.0, I13.0, I13.2 |
| Cerebrovascular disease | ICD-9-CM diagnosis codes: 430-438  ICD-10-CM diagnosis codes: I60-I63, I65-I69, G45, G46 |
| Ischemic stroke | ICD-9-CM diagnosis codes: 433, 434  ICD-10-CM diagnosis codes: I63, I65, I66 |
| Hemorrhagic stroke | ICD-9-CM diagnosis codes: 430-432  ICD-10-CM diagnosis codes: I60-I62 |
| Peripheral vascular disease | ICD-9-CM diagnosis codes: 440.2, 440.4, 443.81, 443.9  ICD-10-CM diagnosis codes: I70.2, I70.92, I79.8, I73.9 |
| Diabetes mellitus | ICD-9-CM diagnosis codes: 250  ICD-10-CM diagnosis codes: E08-E11, E13 |
| Hyperlipidemia | ICD-9-CM diagnosis codes: 272  ICD-10-CM diagnosis codes: E71.30, E75.21, E75.22, E75.24, E75.3, E75.5, E75.6, E77, E78.0-E78.6, E78.70, E78.79, E78.8, E78.9, E88.1, E88.2, E88.9 |
| Thyroid disease | ICD-9-CM diagnosis codes: 242, 244, 245  ICD-10-CM diagnosis codes: E01-E03, E05, E06, E89.0 |
| Pneumonia | ICD-9-CM diagnosis codes: 480-486  ICD-10-CM diagnosis codes: J12-J17, J18.0-J18.2, A481, A37.91, A22.1, B25.0, B44.0 |
| Influenza | ICD-9-CM diagnosis codes: 487  ICD-10-CM diagnosis codes: J09-J11 |
| Acute bronchitis | ICD-9-CM diagnosis codes: 466  ICD-10-CM diagnosis codes: J18.9, J20.8, J20.9, J21 |
| Chronic liver disease | ICD-9-CM diagnosis codes: 070.2, 070.3, 070.41, 070.44, 070.51, 070.54, V02.61, V02.62, 571.0-571.6  ICD-10-CM diagnosis codes: B16, B17, B18.0-B18.2, B19.1, B19.2, Z22.51, Z22.52, K70, K73, K74.0, K74.3-K74.6, K75.4, K75.81 |
| Gastritis or peptic ulcer disease | ICD-9-CM diagnosis codes: 531-535, 578.0, 578.1, 578.9  ICD-10-CM diagnosis codes: K25-K29, K31.82, K52.81, K92.0-K92.2 |
| Chronic kidney disease | ICD-9-CM diagnosis codes: 403.00, 403.01, 403.10, 403.11, 403.90, 403.91, 404.00-404.03, 404.10-404.13, 404.90-404.93, 585, V45.1, V56.0, V56.8  ICD-10-CM diagnosis codes: I12.0, I13.11, I13.2, N18, Z99.2, Z49.31, Z49.32 |
| Rheumatoid arthritis or osteoarthritis | ICD-9-CM diagnosis codes: 714, 715, 720  ICD-10-CM diagnosis codes: M05-M08, M12.0, M15-M19, M32, M33, M34.0, M34.1, M34.9, M35.0, M35.8, M35.9, M45, M46.0, M46.1, M46.8, M46.9, M49 |
| Osteoporosis | ICD-9-CM diagnosis codes: 733.0  ICD-10-CM diagnosis codes: M81 |
| Gout | ICD-9-CM diagnosis codes: 274  ICD-10-CM diagnosis codes: M10, M1A.0, M1A.2-M1A.9, N20.0 |
| Any cancer other than lung cancer | ICD-9-CM diagnosis codes: 140-161, 163-208  ICD-10-CM diagnosis codes: C00-C26, C30-C32, C37-C41, C43-C58, C60-C86, C88, C90-C96, C4A, C7A, C7B, D03, D45, Z51.12 |
| Lung cancer | ICD-9-CM diagnosis codes: 162  ICD-10-CM diagnosis codes: C33, C34 |
| Dementia | ICD-9-CM diagnosis codes: 290.0, 290.3, 291.2, 331.0-331.2, 290.10-290.13, 290.20, 290.21, 290.40-290.43, 294.10, 294.11, 331.82  ICD-10-CM diagnosis codes: F01, F02, F03.90, F05, F10.27, G30, G31.01, G31.09, G31.1, G31.83 |
| Seizure | ICD-9-CM diagnosis codes: 345, 780.3  ICD-10-CM diagnosis codes: G40, R56 |
| Depressive disorder | ICD-9-CM diagnosis codes: 296.2, 296.3, 298.0, 300.4, 309.0, 309.1, 293.83, 296.90, 309.28, 296.82, 311  ICD-10-CM diagnosis codes: F32, F33, F34.1, F43.21, F43.23, F06.3, F39 |
| Anxiety disorder | ICD-9-CM diagnosis codes: 300.0-300.3,300.5-300.9  ICD-10-CM diagnosis codes: F40-F42, F44, F45.0-F45.2, F45.8, F45.9, F48.1, F48.8, F48.9, F68.11, F68.8, F99, R45.2, R45.5, R45.6 |
| Psychotic disorder | ICD-9-CM diagnosis codes: 295, 297-299, 290.8, 290.9, 780.1  ICD-10-CM diagnosis codes: F20, F22, F23, F24, F25, F28, F29, F32.3, F33.3, F44.89, F84.0, F84.3, F84.5, F84.8, F84.9, R44.0, R44.2, R44.3 |
| Bipolar disorder | ICD-9-CM diagnosis codes: 296.0, 296.1, 296.4-296.9  ICD-10-CM diagnosis codes: F30, F31, F32.8, F33.8, F34.8, F34.9, F39 |
|  | Healthcare services |
| Pneumococcal vaccination | ICD-9 CM diagnosis codes: V03.82  Taiwan health insurance service claims codes: K000492206 |
| Influenza vaccination | ICD-9 CM diagnosis codes: V04.7, V04.8  Taiwan health insurance service claims codes: J000113277, K000453265, K000453277, K000523206, K000523265, K000706206, K000889206, X000092206, X000090238, X000090221, X000091221, J000113265, K000901206, J000138206, K001036206, K001126206, X000209206  Case type: D2  Func seq no: 01, 1, IC01 |

^a^Based on any diagnosis or procedure positions or health insurance services records in the outpatient or inpatient claims.

^b^https://www.nhi.gov.tw/query/query2.aspx Accessed on February 26, 2023.

**Table S6.** Anatomical Therapeutic Chemical (ATC) classification system codes used to identify medication use at baseline

| Medications | ATC codes |
| --- | --- |
| ACEI or ARB | C09 |
| Selective β_1_ blockers | C07AB, C07BB, C07CB, C07DB, C07EB, C07FB, C07FX03, C07FX04, C07FX05 |
| Non-selective β_1_ blockers | C07AA, C07AG, C07BA, C07BG, C07CA, C07CG, C07DA, C07EA, C07FX01, C07FX02, C07FX06 |
| Calcium channel blockers | C08 |
| Diuretics | C03, C07B, C07C, C07D, C08G |
| Other anti-hypertensive agents | C02 |
| Nitrates | C01DA |
| Anti-arrhythmic agents | C01B |
| Digoxin | C01AA |
| Aspirin | B01AC06, N02BA01 |
| Clopidogrel | B01AC04 |
| Warfarin | B01AA03 |
| Direct thrombin or factor Xa inhibitors | B01AE, B01AF |
| Statins | C10AA |
| Fibrates | C10AB |
| Urate-lowering agents | M04A |
| Febuxostat | M04AA03 |
| Insulin | A10A |
| Metformin | A10BA02, A10BD02, A10BD03, A10BD05, A10BD07, A10BD08, A10BD10, A10BD11, A10BD13-A10BD18, A10BD20, A10BD22, A10BD23, A10BD25, A10BD26 |
| Sulfonylurea | A10BB, A10BD01, A10BD02, A10BD04, A10BD06 |
| Glinides | A10BX02, A10BX03, A10BX08, A10BD14 |
| Thiazolidinedione | A10BG, A10BD03-A10BD06, A10BD09, A10BD12, A10BD26 |
| Alpha-glucosidase inhibitors | A10BF, A10BD17 |
| Dipeptidyl peptidase-4 inhibitors | A10BH, A10BD07-A10BD13, A10BD18, A10BD19, A10BD21, A10BD22, A10BD24, A10BD25 |
| Sodium-glucose cotransporter 2 Inhibitors | A10BK, A10BD15, A10BD16, A10BD19-A10BD21, A10BD23-A10BD25 |
| Glucagon-like peptide-1 receptor agonists | A10BJ |
| Thyroid-therapy drugs | H03 |
| Inhaled short-acting bronchodilators | SABA: R03AC02-R03AC10, R03AC15-R03AC17  SAMA: R03BB01, R03BB02  SABA/SAMA FDC: R03AL01, R03AL02 |
| Inhaled long-acting bronchodilators | LABA: R03AC12-R03AC14, R03AC18, R03AC19  LAMA: R03BB04-R03BB08  LABA/LAMA/ICS FDC: R03AL08, R03AL09, R03AL11, R03AL12 |
| ICS | ICS: R03BA  LABA/LAMA/ICS FDC: R03AL08, R03AL09, R03AL11, R03AL12 |
| Systemic bronchodilators | Oral β_2_ agonists: R03CC02-R03CC14, R03CC53, R03CC63, R03CC91  Oral xanthines: R03DA04, R03DA05 |
| Systemic corticosteroids | H02AB, H02BX |
| Antibiotics | J01 |
| Histamine 2 antagonists or proton pump inhibitors | A02BA, A02BC |
| COX-2 selective NSAID | M01AH |
| COX-2 nonselective NSAID | M01AA, M01AB, M01AC, M01AE, M01AG, M01AX |
| Anti-epileptics | N03 |
| Anti-depressants: | N06A |
| Anxiolytics | N05B |
| Hypnotics | N05C |
| Anti-psychotics | N05A |

ACEI, angiotensin converting enzyme inhibitors; ARB, angiotensin II receptor blockers; COX-2, cyclooxygenase-2; FDC, fixed-dose combinations; ICS, inhaled corticosteroid; LABA, long-acting β_2_ agonists; LAMA, long-acting muscarinic antagonists. NSAID, non-steroidal anti-inflammatory drugs; SABA, short-acting β_2_ agonists; SAMA, short-acting muscarinic antagonists.

^a^Based on outpatient or inpatient pharmacy dispensing claims.

**Table S7.** Measurement of the 11 clinical parameters at baseline^a^

| Parameters | Acceptable range^b^ |
| --- | --- |
| Variables from the NHI Laboratory Database | |
| Laboratory test |  |
| Eosinophil, % | No restriction |
| C-reactive protein, mg/dL | 0-50 mg/dL |
| LDL-cholesterol, mg/dL | >15 mg/dL |
| HbA1c, % | 4-20% |
| GFR, mL/min | 0-200 mL/min/1.73 m^2^ |
| Serum creatinine, mg/dL | 0.2-20 mg/dL (used for calculating eGFR, see the formula below) |
| Variables from the COPD P4P Database | |
| Lung function test and respiratory symptoms |  |
| Predicted post-dose FEV_1_, % | 10-140% |
| Post-dose FEV_1_/FVC, % | 10-100% |
| CAT score | No restriction |
| Blood pressure and health behavior |  |
| SBP, mmHg | No restriction |
| BMI, kg/m^2^ | 100-200 cm for height  40-200 kg for weight |
| Smoking status | No restriction |
| Note: eGFR was calculated based on information on age, sex, and serum creatinine measured within 0 to 365 days before cohort entry derived from the CKD-EPI formula.[27]  eGFR (mL/min/1.73 m^2^) = 141 × min (Scr /κ, 1)^α^ × max(Scr /κ, 1)^-1.209^ × 0.993^Age^ × 1.018 [if female] × 1.159 [if black]  κ is 0.7 for females and 0.9 for males,  α is -0.329 for females and -0.411 for males,  min indicates the minimum of serum creatinine /κ or 1, and max indicates the maximum of serum creatinine /κ or 1. | |

BMI, body mass index; CAT, COPD Assessment Test; CKD-EPI, Chronic Kidney Disease Epidemiology Collaboration; COPD, chronic obstructive pulmonary disease; eGFR, estimated glomerular filtration rate; FEV_1_, forced expiratory volume in one second; FVC, forced vital capacity; GFR, glomerular filtration rate; HbA1c, glycated hemoglobin; LDL, low-density lipoprotein; NHI, National Health Insurance; P4P, pay for performance; SBP, systolic blood pressure; Scr, serum creatinine.

^a^If there were more than two values available within 365 days before cohort entry, the last record was used for the analysis.

^b^The records of each parameter were excluded if the values were beyond the acceptable range.

**Table S8.** Summary of subgroup analyses^a^

| Rationale or purposes | Approaches |
| --- | --- |
| To evaluate potential effect measure modification by patient characteristic | We conducted subgroup analyses by   - Age at cohort entry (≥65 and <65 years) - Sex (male and female) - COPD duration^b^ (<1 year, 1-2 years, and >2 years) - History of hospitalized COPD exacerbations^c^ (with and without corresponding diagnosis records) - History of cardiovascular diseases^d^ (with and without corresponding diagnosis records) |
| To assess whether the association was different comparing individual LABA/LAMA FDC with individual LABA/ICS FDC | We conducted 15 pair-wise comparisons separately, including   - IND/GLY versus FOR/BEC, FOR/BUD, FOR/FLU, SAL/FLU, and VIL/FLU. - OLO/TIO versus FOR/BEC, FOR/BUD, FOR/FLU, SAL/FLU, and VIL/FLU. - VIL/UME versus FOR/BEC, FOR/BUD, FOR/FLU, SAL/FLU, and VIL/FLU. |
| To explore potential treatment duration-response relation for LABA/LAMA FDC | We separately estimated HRs   - From cohort entry to 90 days after cohort entry for all eligible patients - From 91 to 180 days after cohort entry for patients treated for at least 90 days - From 181 to 365 days after cohort entry for patients treated for at least 180 days - From 366 days to end of the follow-up after cohort entry for patient treated for at least 365 days |

COPD, chronic obstructive pulmonary disease; FDC, fixed-dose combination; FOR/BEC, formoterol/beclomethasone; FOR/BUD, formoterol/budesonide; FOR/FLU, formoterol/fluticasone; HR, hazards ratio; ICS, Inhaled corticosteroids; IND/GLY, indacaterol/glycopyrronium; LABA, long-acting β_2_ agonists; LAMA, long-acting muscarinic antagonists; OLO/TIO, olodaterol/tiotropium; SAL/FLU, salmeterol/fluticasone; VIL/FLU, vilanterol/fluticasone; VIL/UME, vilanterol/umeclidinium.

^a^We re-estimated PS and re-matched patients in each patient subgroup.[28,29]

^b^COPD duration was defined as the duration from the first recorded date of COPD diagnosis (looking back until 2014/01/01) to the cohort entry date.

^c^History of hospitalized COPD exacerbations was defined as having hospitalized COPD exacerbations within 365 days before cohort entry based on any diagnosis positions in the inpatient claims.

^d^History of cardiovascular diseases was defined as having the following cardiovascular diseases within 365 days before cohort entry based on any diagnosis or procedure positions or health services records in the outpatient and inpatient claims, including hypertension, ischemic heart disease or angina, myocardial infarction, coronary revascularization, cardiac arrhythmia, congestive heart failure, cerebrovascular disease, ischemic stroke, hemorrhagic strike, peripheral vascular disease, diabetes mellitus, and hyperlipidemia.

**Table S9.** Patient characteristics of the eligible cohort before and after PS matching

|  | Before PS matching (n=99,506) | | | After PS matching (n=75,926) | | |
| --- | --- | --- | --- | --- | --- | --- |
|  | LABA/LAMA FDC | LABA/ICS FDC | Standardized difference | LABA/LAMA FDC | LABA/ICS FDC | Standardized difference |
|  | n=61,221 | n=38,285 |  | n=48,864 | n=27,062 |  |
|  |  |  |  | n27,062^a^ | n=27,062^a^ |  |
|  | Demographics | | | | | |
| Age, years, mean (SD) | 70.23 (11.48) | 68.64 (12.71) | 0.132 | 68.63 (11.68) | 68.69 (12.50) | -0.005 |
| Male, n (%) | 52,023 (84.98) | 25,032 (65.38) | 0.466 | 20,580 (76.05) | 20,893 (77.20) | -0.027 |
| COPD duration, days, mean (SD)^b^ | 680.71 (730.58) | 657.27 (706.95) | 0.033 | 652.08 (725.58) | 659.15 (708.78) | -0.010 |
| Calendar year of the cohort entry date, n (%) | | | | | | |
| 2017 | 14,175 (23.15) | 10,680 (27.90) | -0.109 | 7,251 (26.79) | 7,176 (26.52) | 0.006 |
| 2018 | 16,094 (26.29) | 9,991 (26.10) | 0.004 | 7,142 (26.39) | 7,069 (26.12) | 0.006 |
| 2019 | 16,486 (26.93) | 9,869 (25.78) | 0.026 | 6,955 (25.70) | 7,083 (26.17) | -0.011 |
| 2020 | 14,466 (23.63) | 7,745 (20.23) | 0.082 | 5,714 (21.11) | 5,734 (21.19) | -0.002 |
|  | Comorbidities, n (%) | | | | | |
| Hypertension | 35,598 (58.15) | 22,453 (58.65) | -0.010 | 15,607 (57.67) | 15,629 (57.75) | -0.002 |
| Ischemic heart disease or angina | 16,541 (27.02) | 9,965 (26.03) | 0.022 | 6,992 (25.84) | 7,037 (26.00) | -0.004 |
| Myocardial infarction | 1,991 (3.25) | 1,133 (2.96) | 0.017 | 769 (2.84) | 757 (2.80) | 0.003 |
| Coronary revascularization | 1,388 (2.27) | 819 (2.14) | 0.009 | 578 (2.14) | 574 (2.12) | 0.001 |
| Cardiac dysrhythmia | 10,215 (16.69) | 6,135 (16.02) | 0.018 | 4,224 (15.61) | 4,198 (15.51) | 0.003 |
| Congestive heart failure | 9,932 (16.22) | 6,333 (16.54) | -0.009 | 4,113 (15.20) | 4,134 (15.28) | -0.002 |
| Cerebrovascular disease | 9,440 (15.42) | 6,141 (16.04) | -0.017 | 4,019 (14.85) | 4,107 (15.18) | -0.009 |
| Ischemic stroke | 4,638 (7.58) | 2,840 (7.42) | 0.006 | 1,906 (7.04) | 1,926 (7.12) | -0.003 |
| Hemorrhagic stroke | 1,220 (1.99) | 947 (2.47) | -0.033 | 562 (2.08) | 564 (2.08) | -0.001 |
| Peripheral vascular disease | 1,828 (2.99) | 1,126 (2.94) | 0.003 | 777 (2.87) | 791 (2.92) | -0.003 |
| Diabetes mellitus | 17,051 (27.85) | 11,164 (29.16) | -0.029 | 7,605 (28.10) | 7,574 (27.99) | 0.003 |
| Hyperlipidemia | 22,042 (36.00) | 14,589 (38.11) | -0.044 | 10,196 (37.68) | 10,110 (37.36) | 0.007 |
| Thyroid disease | 2,099 (3.43) | 1,540 (4.02) | -0.031 | 938 (3.47) | 937 (3.46) | <0.001 |
| Pneumonia | 9,273 (15.15) | 5,927 (15.48) | -0.009 | 3,792 (14.01) | 3,817 (14.10) | -0.003 |
| Influenza | 4,047 (6.61) | 2,557 (6.68) | -0.003 | 1,762 (6.51) | 1,755 (6.49) | 0.001 |
| Acute bronchitis | 29,994 (48.99) | 19,821 (51.77) | -0.056 | 13,451 (49.70) | 13,480 (49.81) | -0.002 |
| Chronic liver disease | 8,517 (13.91) | 5,149 (13.45) | 0.013 | 3,687 (13.62) | 3,674 (13.58) | 0.001 |
| Gastritis or peptic ulcer disease | 24,025 (39.24) | 15,298 (39.96) | -0.015 | 10,681 (39.47) | 10,647 (39.34) | 0.003 |
| Chronic kidney disease | 9,554 (15.61) | 5,549 (14.49) | 0.031 | 3,720 (13.75) | 3,804 (14.06) | -0.009 |
| Rheumatoid arthritis or osteoarthritis | 14,735 (24.07) | 10,564 (27.59) | -0.081 | 7,092 (26.21) | 6,990 (25.83) | 0.009 |
| Osteoporosis | 2,611 (4.26) | 2,070 (5.41) | -0.053 | 1,243 (4.59) | 1,257 (4.64) | -0.002 |
| Gout | 10,162 (16.60) | 6,071 (15.86) | 0.020 | 4,406 (16.28) | 4,490 (16.59) | -0.008 |
| Any cancer other than lung cancer | 8,028 (13.11) | 3,822 (9.98) | 0.098 | 2,725 (10.07) | 2,739 (10.12) | -0.002 |
| Lung cancer | 4,065 (6.64) | 1,317 (3.44) | 0.147 | 949 (3.51) | 991 (3.66) | -0.008 |
| Dementia | 3,897 (6.37) | 3,014 (7.87) | -0.059 | 1,803 (6.66) | 1,780 (6.58) | 0.003 |
| Seizure | 334 (0.55) | 312 (0.81) | -0.033 | 172 (0.64) | 170 (0.63) | 0.001 |
| Depressive disorder | 4,116 (6.72) | 2,931 (7.66) | -0.036 | 1,983 (7.33) | 1,939 (7.17) | 0.006 |
| Anxiety disorder | 8,531 (13.93) | 6,172 (16.12) | -0.061 | 4,159 (15.37) | 4,112 (15.19) | 0.005 |
| Psychotic disorder | 871 (1.42) | 610 (1.59) | -0.014 | 418 (1.54) | 415 (1.53) | 0.001 |
| Bipolar disorder | 980 (1.60) | 670 (1.75) | -0.012 | 448 (1.66) | 457 (1.69) | -0.003 |
|  | Medication use, n (%) | | | | | |
| ACEI or ARB | 26,268 (42.91) | 16,658 (43.51) | -0.012 | 11,572 (42.76) | 11,635 (42.99) | -0.005 |
| Selective β_1_ blockers | 13,683(22.35) | 8,378(21.88) | 0.011 | 5,850 (21.62) | 5,861 (21.66) | -0.001 |
| Non-selective β_1_ blockers | 9,672(15.80) | 6,041(15.78) | 0.001 | 4,147 (15.32) | 4,183 (15.46) | -0.004 |
| Calcium channel blockers | 24,041 (39.27) | 14,861 (38.82) | 0.009 | 10,176 (37.60) | 10,206 (37.71) | -0.002 |
| Diuretics | 17,530 (28.63) | 11,038 (28.83) | -0.004 | 7,093 (26.21) | 7,189 (26.56) | -0.008 |
| Other anti-hypertensive agents | 6,798 (11.10) | 3,729 (9.74) | 0.045 | 2,632 (9.73) | 2,692 (9.95) | -0.007 |
| Nitrates | 11,198 (18.29) | 6,741 (17.61) | 0.018 | 4,656 (17.20) | 4,700 (17.37) | -0.004 |
| Anti-arrhythmic agents | 5,677 (9.27) | 3,304 (8.63) | 0.023 | 2,166 (8.00) | 2,199 (8.13) | -0.004 |
| Digoxin | 1,772 (2.89) | 1,178 (3.08) | -0.011 | 724 (2.68) | 734 (2.71) | -0.002 |
| Aspirin | 18,324 (29.93) | 10,865 (28.38) | 0.034 | 7,781 (28.75) | 7,774 (28.73) | 0.001 |
| Clopidogrel | 5,889 (9.62) | 3,565 (9.31) | 0.011 | 2,390 (8.83) | 2,423 (8.95) | -0.004 |
| Warfarin | 1,186 (1.94) | 723 (1.89) | 0.004 | 477 (1.76) | 458 (1.69) | 0.005 |
| Direct thrombin or factor Xa inhibitors | 3,298 (5.39) | 1,928 (5.04) | 0.016 | 1,302 (4.81) | 1,282 (4.74) | 0.003 |
| Statins | 16,761 (27.38) | 10,797 (28.20) | -0.018 | 7,471 (27.61) | 7,471 (27.61) | 0 |
| Fibrates | 2,452 (4.01) | 1,639 (4.28) | -0.014 | 1,173 (4.33) | 1,208 (4.46) | -0.006 |
| Urate-lowering agents | 10,130 (16.55) | 5,852 (15.29) | 0.034 | 4,212 (15.56) | 4,338 (16.03) | -0.013 |
| Febuxostat | 2,981 (4.87) | 1,690 (4.41) | 0.022 | 1,195 (4.42) | 1,204 (4.45) | -0.002 |
| Insulin | 6,016 (9.83) | 3,726 (9.73) | 0.003 | 2,259 (8.35) | 2,332 (8.62) | -0.010 |
| Metformin | 10,245 (16.73) | 6,639 (17.34) | -0.016 | 4,569 (16.88) | 4,550 (16.81) | 0.002 |
| Sulfonylurea | 6,437 (10.51) | 3,984 (10.41) | 0.004 | 2,786 (10.29) | 2,777 (10.26) | 0.001 |
| Glinides | 1,634 (2.67) | 1,090 (2.85) | -0.011 | 652 (2.41) | 681 (2.52) | -0.007 |
| Thiazolidinedione | 1,721 (2.81) | 1,024 (2.67) | 0.008 | 715 (2.64) | 721 (2.66) | -0.001 |
| Alpha-glucosidase inhibitors | 1,703 (2.78) | 1,046 (2.73) | 0.003 | 702 (2.59) | 699 (2.58) | 0.001 |
| Dipeptidyl peptidase-4 inhibitors | 7,013 (11.46) | 4,471 (11.68) | -0.007 | 2,988 (11.04) | 3,009 (11.12) | -0.002 |
| Sodium-glucose cotransporter 2 Inhibitors | 1,160 (1.89) | 740 (1.93) | -0.003 | 532 (1.97) | 516 (1.91) | 0.004 |
| Glucagon-like peptide-1 receptor agonists | 167 (0.27) | 114 (0.30) | -0.005 | 74 (0.27) | 64 (0.24) | 0.007 |
| Thyroid-therapy drugs | 1,704 (2.78) | 1,266 (3.31) | -0.030 | 763 (2.82) | 743 (2.75) | 0.004 |
| Inhaled short-acting bronchodilators | 27,375 (44.72) | 15,665 (40.92) | 0.077 | 10,445 (38.60) | 10,646 (39.34) | -0.015 |
| Inhaled long-acting bronchodilators | 26,359 (43.06) | 10,374 (27.10) | 0.339 | 4,141 (15.30) | 4,122 (15.23) | 0.002 |
| ICS | 1,764 (2.88) | 8,454 (22.08) | -0.607 | 1,763 (6.51) | 1,862 (6.88) | -0.015 |
| Systemic bronchodilators | 44,762 (73.12) | 28,903 (75.49) | -0.054 | 20,554 (75.95) | 20,549 (75.93) | <0.001 |
| Systemic corticosteroids | 35,421 (57.86) | 23,697 (61.90) | -0.082 | 16,055 (59.33) | 16,069 (59.38) | -0.001 |
| Antibiotics | 46,719 (76.31) | 29,552 (77.19) | -0.021 | 20,450 (75.57) | 20,442 (75.54) | 0.001 |
| Histamine 2 antagonists or proton pump inhibitors | 35,075 (57.29) | 22,146 (57.85) | -0.011 | 15,345 (56.70) | 15,214 (56.22) | 0.010 |
| COX-2 selective NSAIDs | 11,263 (18.40) | 7,712 (20.14) | -0.044 | 5,137 (18.98) | 5,151 (19.03) | -0.001 |
| COX-2 nonselective NSAIDs | 42,024 (68.64) | 26,906 (70.28) | -0.036 | 19,031 (70.32) | 19,084 (70.52) | -0.004 |
| Anti-epileptics | 8,652 (14.13) | 6,179 (16.14) | -0.056 | 4,058 (15.00) | 4,059 (15.00) | <0.001 |
| Anti-depressants: | 8,414 (13.74) | 5,819 (15.20) | -0.041 | 3,933 (14.53) | 3,890 (14.37) | 0.005 |
| Anxiolytics | 27,075 (44.23) | 17,805 (46.51) | -0.046 | 12,165 (44.95) | 12,199 (45.08) | -0.003 |
| Hypnotics | 14,175 (23.15) | 9,281 (24.24) | -0.026 | 6,190 (22.87) | 6,162 (22.77) | 0.002 |
| Anti-psychotics | 10,350 (16.91) | 6,792 (17.74) | -0.022 | 4,446 (16.43) | 4,390 (16.22) | 0.006 |
|  | Health services, mean (SD) | | | | | |
| Pneumococcal or influenza vaccination | 25,457 (41.58) | 15,797 (41.26) | 0.007 | 11,040 (40.80) | 11,122 (41.10) | -0.006 |
| No. of any outpatient visit | 39.66 (26.04) | 40.05 (26.41) | -0.015 | 39.32 (26.41) | 39.29 (26.24) | 0.001 |
| No. of outpatient visit due to COPD | 5.23 (6.54) | 4.40 (5.83) | 0.134 | 4.34 (5.77) | 4.43 (5.81) | -0.016 |
| No. of outpatient visit due to cardiovascular diseases^c^ | 11.13 (12.40) | 11.34 (12.76) | -0.017 | 10.91 (12.30) | 10.99 (12.45) | -0.006 |
| No. of any hospitalization | 0.82 (1.56) | 0.73 (1.55) | 0.052 | 0.65 (1.32) | 0.66 (1.48) | -0.008 |
| No. of hospitalization due to COPD | 0.28 (0.75) | 0.23 (0.69) | 0.077 | 0.18 (0.58) | 0.19 (0.65) | -0.009 |
| No. of hospitalizations due to cardiovascular disease^c^ | 0.52 (1.12) | 0.48 (1.11) | 0.035 | 0.42 (0.97) | 0.43 (1.06) | -0.007 |

ACEI, angiotensin converting enzyme inhibitors; ARB, angiotensin receptor blockers; COPD, chronic obstructive pulmonary disease; COX-2, cyclooxygenase-2; FDC, fixed-dose combinations; ICS, inhaled corticosteroids; LABA, long-acting β_2_ agonists; LAMA, long-acting muscarinic antagonists; NSAID, non-steroidal anti-inflammatory drugs; PS, propensity score; SD, standard deviation.

^a^One randomly sampled LABA/LAMA FDC initiator versus one LABA/ICS FDC initiator in each matched subset.

^b^COPD duration was defined as the duration from the first recorded date of COPD diagnosis (looking back until 2014/01/01) to the cohort entry date.

^c^Cardiovascular disease include hypertension, ischemic heart disease or angina, myocardial infarction, coronary revascularization, cardiac dysrhythmia, congestive heart failure, cerebrovascular disease, ischemic stroke, hemorrhagic stroke, transient ischemic attack, peripheral vascular disease, diabetes mellitus, and hyperlipidemia.

**Table S10.** Number of patients and events, follow-up duration, incidence rate, and risk of pneumonia comparing LABA/LAMA FDC with LABA/ICS FDC before and after PS matching

|  | Before PS matching (n=99,506) | | After PS matching (n=75,926) | |
| --- | --- | --- | --- | --- |
|  | LABA/LAMA FDC | LABA/ICS FDC | LABA/LAMA FDC | LABA/ICS FDC |
|  | n=61,221 | n=38,285 | n=48,864 | n=27,062 |
| Number of events | 1,222 | 716 | 848 | 446 |
| Mean follow-up days (SD) | 255.32 (276.97) | 162.06 (183.53) | 247.09 (271.60) | 156.98 (176.32) |
| Incidence rate (95% CI)^a^ | 28.55 (27.00-30.20) | 42.15 (39.17-45.35) | 23.64 (21.46-26.04) | 38.35 (34.95-42.07) |
| HR (95% CI)^b^ | 0.74 (0.68-0.82) | Reference | 0.65 (0.58-0.74) | Reference |

CI, confidence interval; FDC, fixed-dose combinations; HR, hazards ratio; ICS, inhaled corticosteroids; LABA, long-acting β_2_ agonists; LAMA, long-acting muscarinic antagonists; PS, propensity score; SD, standard deviation.

^a^The unit of incidence rate was per 1,000 person-years. The incidence rate after PS matching was weighted by the inverse of the matching ratio.

^b^The HR after PS matching was stratified on the inverse of the matching ratio.

**Table S11.** Number of patients and events, follow-up duration, incidence rate, and risk of composite cardiovascular events comparing LABA/LAMA FDC with LABA/ICS FDC before and after PS matching, by intention-to-treat approach

|  | Before PS matching (n=99,506) | | After PS matching (n=75,926) | |
| --- | --- | --- | --- | --- |
|  | LABA/LAMA FDC | LABA/ICS FDC | LABA/LAMA FDC | LABA/ICS FDC |
|  | n=61,221 | n=38,285 | n=48,864 | n=27,062 |
| Number of events | 2,329 | 1,472 | 1,852 | 1,041 |
| Mean follow-up days (SD) | 635.44 (414.91) | 683.51 (423.88) | 650.53 (415.86) | 676.06 (421.95) |
| Incidence rate^a^ (95% CI)^a^ | 21.87 (21.00-22.77) | 20.55 (19.52-21.62) | 20.42 (19.21-21.71) | 20.78 (19.56-22.08) |
| HR (95% CI)^b^ | 1.05 (0.98,1.12) | Reference | 0.97 (0.90-1.05) | Reference |

CI, confidence interval; FDC, fixed-dose combinations; HR, hazards ratio; ICS, inhaled corticosteroids; LABA, long-acting β_2_ agonists; LAMA, long-acting muscarinic antagonists; PS, propensity score; SD, standard deviation.

^a^The unit of incidence rate was per 1,000 person-years. The incidence rate after PS matching was weighted by the inverse of the matching ratio.

^b^The HR after PS matching was stratified on the inverse of the matching ratio.

**Table S12.** Availability of the clinical parameters at baseline in the eligible cohort

| No. (%) of patients with information | LABA/LAMA FDC | LABA/ICS FDC | Total |
| --- | --- | --- | --- |
|  | n=61,221 | n=38,285 | n=99,506 |
| ≥ One clinical parameter, overall | 52,257 (85.36) | 30,768 (80.37) | 83,025 (83.44) |
|  | Variables from the NHI Laboratory Database | | |
| ≥ One test result | 50,878 (83.11) | 30,551 (79.80) | 81,429 (81.83) |
| Eosinophil | 36,220 (59.16) | 21,091 (55.09) | 57,311 (57.60) |
| C-reactive protein | 22,635 (36.97) | 12,930 (33.77) | 35,565 (35.74) |
| LDL-cholesterol | 27,937 (45.63) | 17,492 (45.69) | 45,429 (45.65) |
| HbA1c | 24,915 (40.70) | 15,337 (40.06) | 40,252 (40.45) |
| GFR or eGFR | 48,925 (79.92) | 28,795 (75.21) | 77,720 (78.11) |
|  | Variables from the COPD P4P Database | | |
| ≥ One test result | 6,812 (11.13) | 1,088 (2.84) | 7,900 (7.94) |
| Predicted post-dose FEV_1_ predicted | 6,374 (10.41) | 1,037 (2.71) | 7,411 (7.45) |
| Post-dose FEV_1_/FVC | 6,402 (10.46) | 1,033 (2.70) | 7,435 (7.47) |
| CAT score | 3,585 (5.86) | 563 (1.47) | 4,148 (4.17) |
| SBP | 3,584 (5.85) | 558 (1.46) | 4,142 (4.16) |
| BMI | 3,542 (5.79) | 550 (1.44) | 4,092 (4.11) |
| Smoking status | 3,584 (5.85) | 558 (1.46) | 4,142 (4.16) |

BMI, body mass index; CAT, COPD Assessment Test; COPD, chronic obstructive pulmonary disease; eGFR, estimated glomerular filtration rate; FDC, fixed-dose combinations; FEV_1_, forced expiratory volume in one second; FVC, forced vital capacity; GFR, glomerular filtration rate; HbA1c, glycated hemoglobin; ICS, inhaled corticosteroids; LABA, long-acting β_2_ agonists; LAMA, long-acting muscarinic antagonists; LDL, low-density lipoprotein; NHI, National Health Insurance; P4P, pay for performance; SBP, systolic blood pressure.

**Table S13.** Clinical parameters of the imputed cohort before and after PS matching^a^

|  | Before PS matching (n=99,506) | | | After PS matching (n=74,970) | | |
| --- | --- | --- | --- | --- | --- | --- |
|  | LABA/LAMA FDC | LABA/ICS FDC | Standardized difference | LABA/LAMA FDC | LABA/ICS FDC | Standardized difference |
|  | n=61,221 | n=38,285 |  | n=48,641 | n=26,329 |  |
|  |  |  |  | n=26,329^b^ | n=26,329^b^ |  |
|  | Variables from the NHI Laboratory Database | | | | | |
| Laboratory test | | | | | | |
| Eosinophil, %, mean (SD) | 2.52 (2.87) | 2.74 (3.11) | -0.074 | 2.69 (3.05) | 2.70 (2.94) | -0.004 |
| C-reactive protein, mg/dL, mean (SD) | 2.50 (4.78) | 2.12 (4.69) | 0.078 | 2.13 (4.60) | 2.18 (4.71) | -0.010 |
| LDL-cholesterol, mg/dL, mean (SD) | 101.35 (32.50) | 103.58 (32.85) | -0.068 | 103.59 (32.65) | 103.16 (32.73) | 0.013 |
| HbA1c, %, mean (SD) | 6.20 (1.18) | 6.19 (1.17) | 0.003 | 6.19 (1.16) | 6.19 (1.17) | -0.001 |
| GFR or eGFR, mL/min, mean (SD) | 78.68 (29.46) | 79.20 (30.02) | -0.018 | 78.84 (29.07) | 78.88 (29.20) | -0.001 |
|  | Variables from the COPD P4P Database | | | | | |
| Lung function test and respiratory symptoms | | | | | | |
| Predicted post-dose FEV_1_, %, mean (SD) | 62.59 (20.95) | 64.29 (21.27) | -0.080 | 64.03 (20.94) | 63.98 (21.20) | 0.002 |
| Post-dose FEV_1_/FVC, %, mean (SD) | 60.14 (11.68) | 61.16 (11.74) | -0.087 | 60.64 (11.66) | 60.64 (11.68) | <0.001 |
| CAT score, mean (SD) | 13.01 (7.06) | 13.34 (7.03) | -0.047 | 13.55 (7.04) | 13.54 (7.01) | 0.002 |
| Blood pressure and health behavior | | | | | | |
| SBP, mmHg, mean (SD) | 132.10 (17.71) | 130.93 (17.66) | 0.066 | 131.65 (17.65) | 131.60 (17.63) | 0.003 |
| BMI, kg/m^2^, mean (SD) | 24.16 (4.10) | 24.42 (4.10) | -0.064 | 24.40 (4.08) | 24.43 (4.08) | -0.005 |
| Smoking status, n (%) |  |  |  |  |  |  |
| Never smoker | 10,808 (17.65) | 13,831 (36.13) | -0.426 | 6,999 (26.59) | 6,766 (25.70) | 0.020 |
| Ex-smoker | 29,324 (47.90) | 14,078 (36.77) | 0.227 | 10,987 (41.73) | 11,095 (42.14) | -0.008 |
| Current smoker | 21,089 (34.45) | 1,0376 (27.1) | 0.160 | 8,340 (31.68) | 8,465 (32.15) | -0.010 |

BMI, body mass index; CAT, COPD Assessment Test; COPD, chronic obstructive pulmonary disease; eGFR, estimated glomerular filtration rate; FDC, fixed-dose combinations; FEV_1_, forced expiratory volume in one second; FVC, forced vital capacity; GFR, glomerular filtration rate; HbA1c, glycated hemoglobin; ICS, inhaled corticosteroids; LABA, long-acting β_2_ agonists; LAMA, long-acting muscarinic antagonists; LDL, low-density lipoprotein; NHI, National Health Insurance; P4P, pay for performance; PS, propensity score; SBP, systolic blood pressure; SD, standard deviation.

^a^One randomly sampled dataset from 10 imputed datasets.

^b^One randomly sampled LABA/LAMA FDC initiator versus one LABA/ICS FDC initiator in each matched subset.

**Table S14.** Number of patients, number of events, and risk of composite cardiovascular events comparing LABA/LAMA FDC versus LABA/ICS FDC before and after PS matching, by patient characteristic^a^

|  | Before PS matching | | | After PS matching^a^ | | | |
| --- | --- | --- | --- | --- | --- | --- | --- |
|  | LABA/LAMA FDC | LABA/ICS FDC | Crude HR (95% CI) | LABA/LAMA FDC | LABA/ICS FDC | HR after PS matching (95% CI)^b^ | P value for test of homogeneity |
|  | Events/Patients | |  | Events/Patients | |  |  |
| Age | | | | | | | |
| ≥65 years old | 858/40,815 | 423/23,231 | 0.84 (0.75-0.94) | 657/31,694 | 286/16,631 | 0.86 (0.74-1.00) | 0.303 |
| <65 years old | 212/20,406 | 103/15,054 | 1.14 (0.90-1.45) | 166/17,131 | 70/10,233 | 1.02 (0.76-1.36) |  |
| Sex | | | | | | | |
| Male | 953/52,023 | 395/25,032 | 0.85 (0.75-0.95) | 731/40,646 | 322/20,832 | 0.84 (0.74-0.97) | 0.426 |
| Female | 117/9,198 | 131/13,253 | 0.94 (0.73-1.21) | 73/6,220 | 113/11,781 | 0.96 (0.71-1.29) |  |
| COPD duration^c^ | | | | | | | |
| <1 year | 467/29,956 | 238/18,780 | 0.95 (0.81-1.11) | 358/24,256 | 165/13,116 | 0.82 (0.68-1.00) | 0.237 |
| 1-2 years | 97/5,659 | 54/3,861 | 0.84 (0.60-1.18) | 73/4,541 | 38/2,536 | 0.75 (0.49-1.14) |  |
| >2 years | 506/25,606 | 234/15,644 | 0.90 (0.77-1.06) | 397/19,897 | 157/11,252 | 1.01 (0.83-1.23) |  |
| History of hospitalized COPD exacerbation^d^ | | | | | | | |
| Yes | 326/12,034 | 166/5,858 | 0.75 (0.62-0.91) | 222/7,378 | 101/3,532 | 0.81 (0.63-1.04) | 0.436 |
| No | 744/49,187 | 360/32,427 | 0.973(0.86-1.11) | 609/41,402 | 264/23,412 | 0.91 (0.78-1.06) |  |
| History of cardiovascular disease^e^ | | | | | | | |
| Yes | 955/46,728 | 486/29,297 | 0.91 (0.81-1.01) | 743/37,163 | 330/20,687 | 0.87 (0.76-1.00) | 0.563 |
| No | 115/14,493 | 40/8,988 | 1.17 (0.81-1.69) | 88/11,694 | 33/6,278 | 0.99 (0.65-1.50) |  |

CI, confidence interval; COPD, chronic obstructive pulmonary disease; FDC, fixed-dose combinations; HR, hazards ratio; ICS, inhaled corticosteroids; LABA, long-acting β_2_ agonists; LAMA, long-acting muscarinic antagonists; PS, propensity score.

^a^We re-estimated PS and re-matched patients in each patient subgroup.

^b^The HR after PS matching was stratified on the inverse of the matching ratio.

^c^COPD duration was defined as the duration from the first recorded date of COPD diagnosis (looking back until 2014/01/01) to the cohort entry date.

^d^History of hospitalized COPD exacerbations was defined as having hospitalized COPD exacerbations within 365 days before cohort entry based on any diagnosis positions in the inpatient claims.

^e^History of cardiovascular diseases was defined as having the following cardiovascular diseases within 365 days before cohort entry based on any diagnosis or procedure positions or health services records in the outpatient and inpatient claims, including hypertension, ischemic heart disease or angina, myocardial infarction, coronary revascularization, cardiac arrhythmia, congestive heart failure, cerebrovascular disease, ischemic stroke, hemorrhagic strike, peripheral vascular disease, diabetes, and hyperlipidemia.

**Table S15.** Number of patients, number of events, and risk of composite cardiovascular events comparing LABA/LAMA FDC versus LABA/ICS FDC before and after PS score matching, by individual LABA/LAMA FDC and LABA/ICS FDC

|  | Before PS matching | | | After PS matching^a^ | | | |
| --- | --- | --- | --- | --- | --- | --- | --- |
|  | LABA/LAMA FDC | LABA/ICS FDC | Crude HR (95% CI) | LABA/LAMA FDC | LABA/ICS FDC | HR after PS matching (95% CI)^b^ | P value for test of homogeneity |
|  | Events/Patients | |  | Events/Patients | |  |  |
| IND/GLY versus | | | | | | | |
| FOR/BEC | 245/15,426 | 147/10,311 | 0.75 (0.61-0.93) | 205/12,910 | 96/6,916 | 0.78 (0.61-1.01) | 0.158 |
| FOR/BUD | 245/15,426 | 104/10,351 | 1.06 (0.84-1.34) | 197/12,964 | 80/7,263 | 0.92 (0.70-1.21) |  |
| FOR/FLU | 245/15,426 | 7/434 | 0.56 (0.26-1.19) | 24/2,015 | 7/428 | 0.37 (0.15-0.90) |  |
| SAL/FLU | 245/15,426 | 194/9,605 | 0.57 (0.47-0.69) | 207/12,606 | 105/6,402 | 0.76 (0.60-0.98) |  |
| VIL/FLU | 245/15,426 | 74/7,584 | 1.17 (0.90-1.52) | 196/13,315 | 57/5,623 | 1.05 (0.77-1.43) |  |
| OLO/TIO versus | | | | | | | |
| FOR/BEC | 353/19,189 | 147/10,311 | 0.89 (0.73-1.08) | 219/11,147 | 93/6,425 | 0.91 (0.70-1.18) | 0.705 |
| FOR/BUD | 353/19,189 | 104/10,351 | 1.26 (1.01-1.57) | 199/11,318 | 77/6,400 | 1.02 (0.77-1.35) |  |
| FOR/FLU | 353/19,189 | 7/434 | 0.67 (0.32-1.42) | 32/1,931 | 7/429 | 0.64 (0.27-1.50) |  |
| SAL/FLU | 353/19,189 | 194/9,605 | 0.67 (0.56-0.80) | 215/10,888 | 105/5,544 | 0.84 (0.65-1.08) |  |
| VIL/FLU | 353/19,189 | 74/7,584 | 1.38 (1.07-1.77) | 190/11,347 | 61/5,196 | 1.02 (0.75-1.38) |  |
| VIL/UME versus | | | | | | | |
| FOR/BEC | 472/26,606 | 147/10,311 | 0.92 (0.76-1.11) | 355/21,061 | 118/8,456 | 0.88 (0.71-1.11) | 0.224 |
| FOR/BUD | 472/26,606 | 104/10,351 | 1.30 (1.05-1.61) | 343/21,344 | 93/8,470 | 0.96 (0.76-1.22) |  |
| FOR/FLU | 472/26,606 | 7/434 | 0.69 (0.33-1.46) | 27/2,130 | 7/431 | 0.53 (0.23-1.24) |  |
| SAL/FLU | 472/26,606 | 194/9,605 | 0.70 (0.59-0.82) | 359/19,905 | 132/7,319 | 0.80 (0.65-0.99) |  |
| VIL/FLU | 472/26,606 | 74/7,584 | 1.43 (1.11-1.82) | 301/19,945 | 65/6,356 | 1.14 (0.86-1.51) |  |

CI, confidence interval; COPD, chronic obstructive pulmonary disease; FDC, fixed-dose combinations; FOR/BEC, formoterol/beclomethasone; FOR/BUD, formoterol/budesonide; FOR/FLU, formoterol/fluticasone; HR, hazards ratio; ICS, Inhaled corticosteroids; IND/GLY, indacaterol/glycopyrronium; LABA, long-acting β_2_ agonists; LAMA, long-acting muscarinic antagonists; OLO/TIO, olodaterol/tiotropium; PS, propensity score; SAL/FLU, salmeterol/fluticasone; VIL/FLU, vilanterol/fluticasone; VIL/UME, vilanterol/umeclidinium.

^a^We re-estimated PS and re-matched patients in each patient subgroup.

^b^The HR after PS matching was stratified on the inverse of the matching ratio.

**Table S16.** Number of patients, number of events, and risk of composite cardiovascular events comparing LABA/LAMA FDC versus LABA/ICS FDC before and after PS matching, by treatment duration^a^

|  | Before PS matching | | | After PS matching^a^ | | | |
| --- | --- | --- | --- | --- | --- | --- | --- |
|  | LABA/LAMA FDC | LABA/ICS FDC | Crude HR (95% CI) | LABA/LAMA FDC | LABA/ICS FDC | HR after PS matching (95% CI)^b^ | P value for test of homogeneity |
|  | Events/Patients | |  | Events/Patients | |  |  |
| 1-90 days | 517/61,221 | 319/38,285 | 1.00 (0.87-1.15) | 411/48,864 | 228/27,062 | 0.96 (0.81-1.14) | 0.218 |
| 91-180 days | 168/38,244 | 96/17,278 | 0.71 (0.55-0.91) | 120/26,328 | 73/12,720 | 0.66 (0.49-0.90) |  |
| 181-365 days | 174/24,359 | 58/8,407 | 0.91 (0.68-1.23) | 111/15,305 | 46/6,396 | 0.90 (0.63-1.29) |  |
| >365 days | 211/13,407 | 53/3,580 | 0.91 (0.67-1.23) | 115/7,439 | 39/2,694 | 0.95 (0.65-1.38) |  |

CI, confidence interval; COPD, chronic obstructive pulmonary disease; FDC, fixed-dose combinations; HR, hazards ratio; ICS, inhaled corticosteroids; LABA, long-acting β_2_ agonists; LAMA, long-acting muscarinic antagonists; PS, propensity score.

^a^We re-estimated PS and re-matched patients in each patient subgroup.

^b^The HR after PS matching was stratified on the inverse of the matching ratio.

**Table S17**. Selected patient characteristics at baseline and cardiovascular outcomes during follow-up of three substantial efficacy trials and our study

|  | Three substantial efficacy trials enrolled in the Yang’s meta-analysis | | | Our cohort study |
| --- | --- | --- | --- | --- |
|  | Wedzicha et al. NEJM. 2016  (FLAME trial, NCT01782326) | Lipson et al. NEJM. 2018  (IMPACT trial, NCT02164513) | Rabe et al. NEJM. 2020  (ETHOS trial, NCT02465567) |  |
| Study arm | Total (n=3,362)  IND/GLY FDC (n=1,680)  SAL/FLU FDC (n=1,682) | Total (n=10,355)  VIL/UME/FLU FDC (n=4,151)  VIL/UME FDC (n=2,070)  VIL/FLU FDC (n=4,134) | Total (n=8,509)  FOR/GLY/BUD FDC (n=4,258)  FOR/GLY FDC (n=2,120)  FOR/BUD FDC (n=2,131) | Before 5:1 variable-ratio PS matching (n=99,506)  LABA/LAMA FDC (n=61,211)  LABA/ICS FDC (n=38,285)  After 5:1 variable-ratio PS matching (n=75,926)  LABA/LAMA FDC (n=48,864)  LABA/ICS FDC (n=27,062) |
|  | Selected patient characteristics at baseline | | | |
| Age, years, mean (SD) | 64.6 (7.8) | 65.3 (8.3) | 64.6 (7.6) | 68.7 (12.1)^a^ |
| Male, n (%) | 2,557 (76.1) | 6,870 (66.3) | 5,081 (59.7) | 41,473 (76.6)^a^ |
| COPD duration, year mean (SD) or n (%) in each category | 7.3 (5.4) | <1 year: 542 (5.2)  ≥1 to <5 years: 3,347 (32.3)  ≥5 to <10 years: 3,424 (33.1)  ≥10 to <15 years: 1,871 (18.1)  ≥15 to <20 years: 695 (6.7)  ≥20 to <25 years: 282 (2.7) | 8.3 (6.2) | 1.8 (2.0)^a^ |
| Predicted post-dose FEV_1_, % mean (SD) or n (%) in each category | 44.1 (9.5) | 45.5 (14.8) | 50% to <80%: 2,427 (28.5)  30% to <50%: 5,151 (60.5)  <30%: 924 (10.9) | 60.7 (11.6)^b^ |
| Exacerbation history within one year, n (%) | One moderate/severe episode: 2,710 (80.6)^c^  ≥Two moderate/severe episodes: 649 (19.3)^c^ | One moderate/severe episode: 4,691 (45.3)^c^  ≥Two moderate/severe episodes: 5,655 (54.6)^c^  ≥One severe episode: 2,671 (25.8)^c^ | One moderate/severe exacerbation: 3,691 (43.4)^c^  ≥Two moderate/severe episodes: 4,810 (56.5)^c^  ≥One severe episode: 1,801 (21.2)^c^ | ≥One severe episode: 10,990 (14.47)^d^ |
| CAT score, mean (SD) | 16.7 (7.0) | 20.1 (6.1) | 19.6 (6.5) | 13.5 (7.0)^b^ |
| ICS use within one year, n (%) | 1,893 (56.3) | 7,359 (71.1) | 6,846 (80.5) | 3,625 (6.7)^a^ |
|  | Cardiovascular outcomes during follow-up | | | |
| Follow-up duration | 52-week ITT follow-up | 52-week ITT follow-up | 52-week ITT follow-up | 215-day on-treatment mean follow-up in the PS matched cohort |
| Outcome definition^e^ | MACE, including non-fatal myocardial infarction, non-fatal stroke, and cardiovascular death^e^ | MACE, including non-fatal myocardial infarction, non-fatal stroke, and cardiovascular death^e^ | MACE, including non-fatal myocardial infarction, non-fatal stroke, and cardiovascular death^e^ | Hospitalized cardiovascular events, including myocardial infarction, angina, heart failure, cardiac dysrhythmia, and ischemic stroke |
| No. of events/no. of patients (%) in the LABA/LAMA or LABA/ICS groups^e^ | 24/1,680 (1.4) vs 21/1,682 (1.2)^e^ | 50/2,070 (2.4) vs 77/4,134 (1.9)^e^ | 45/2,120 (2.1) vs 24/2,131 (1.1)^e^ | 819/488,624 (1.7) vs 361/27,062 (1.3) in the PS matched cohort |
| Incidence rate per 1,000 person-years | Not accountable in the Yang et al’s meta-analysis | Not accountable in the Yang et al’s meta-analysis | Not accountable in the Yang et al’s meta-analysis | 25.22 vs 30.90 in the PS matched cohort |
| Risk estimates (95% CI) | Risk ratio: 1.14 (0.64-2.05) | Risk ratio: 1.30 (0.91-1.84) | Risk ratio: 1.88 (1.15-3.08) | HR after PS matching: 0.89 (0.78-1.01) |
|  | Summary risk across three efficacy trials: 119/5,870 (2.0) vs 122/7,947 (1.5)  Summary risk ratio across three efficacy trials: 1.40 (1.08-1.82) | | |  |

CAT, COPD Assessment Test; CI, confidence interval; COPD, chronic obstructive pulmonary disease; ETHOS, The Efficacy and Safety of Triple Therapy in Obstructive Lung Disease; FDC, fixed-dose combinations; FEV_1_, forced expiratory volume in one second; FLAME, The Effect of Indacaterol Glycopyronium Vs. Fluticasone Salmeterol on COPD Exacerbations; FOR/BUD, formoterol/budesonide; FOR/FLU, formoterol/fluticasone; HR, hazards ratio; ICS, inhaled corticosteroids; IMPACT, The Informing the Pathway of COPD Treatment; IND/GLY, indacaterol/glycopyrronium; LABA, long-acting β_2_ agonists; LAMA, long-acting muscarinic antagonists; MACE, major adverse cardiovascular events; OLO/TIO, olodaterol/tiotropium; PS, propensity score; SAL/FLU, salmeterol/fluticasone; SD, standard deviation; VIL/FLU, vilanterol/fluticasone; VIL/UME, vilanterol/umeclidinium.

^a^Based on data from one randomly sampled LABA/LAMA FDC initiator versus one LABA/ICS FDC initiator in each matched subset (see Table 1 or Table S8).

^b^Based on data from one randomly sampled dataset from 10 imputed datasets (see Table S13).

^c^A moderate exacerbation was defined as an exacerbation leading to treatment with systemic glucocorticoids or antibiotics; a severe exacerbation was defined as an exacerbation leading to hospital admissions, emergency department visits, or death.

^d^A severe exacerbation was defined as an exacerbation leading to hospital admissions.

^e^Outcome definition and no. of events/no. of patients (%) in the three efficacy trials are based on Yang et al’s meta-analysis.

**Figure S1.** Study cohort assembly

| Patients with a COPD diagnosis during 2017/01/01-2020/12/31 (n=1,495,108) | |  | |
| --- | --- | --- | --- |
|  |  |  |  |
|  |  | Excluded due to   - Age ≤40 years or age >100 years on the COPD diagnosis date (n= 220,358) - Ambiguous or missing information on age and sex (n=6) - Patients who did not receive LABA/LAMA FDC or LABA/ICS FDC after the COPD diagnosis date (n=973,178) | |
|  |  |  |  |
|  |  |  |  |
| Patients with COPD who received LABA/LAMA FDC or LABA/ICS FDC (n=281,568) | |  |  |
|  |  |  |  |
|  |  | Excluded due to   - Patients who did not have interactions with the healthcare system within 365 days before cohort entry (n=430) - Patients who received LABA/LAMA FDC or LABA/ICS FDC within 365 days before cohort entry (n=62,950) - Patients who simultaneously received LABA/LAMA FDC and LABA/ICS FDC at cohort entry (n=508) - Patients who simultaneously received LABA, LAMA, and ICS at cohort entry (n=10,290) - Patients who received more than one LABA/LAMA FDC or more than one LABA/ICS FDC at cohort entry (n=93) - Patients who had an asthma diagnosis within 365 days before cohort entry (n=107,677) - Patients who had death records before cohort entry (n=20) - Patients with a cohort entry date on 2020/12/31 (n=94) | |
|  |  |  |  |
|  |  |  |  |
| Potential study population (n=99,506)   - LABA/LAMA FDC initiators (n=61,221)   - IND/GLY (n=15,426)  - OLO/TIO (n=19,189)  - VIL/UME (n=26,606)   - LABA/ICS FDC initiators (n=38,285)   - FOR/BEC (n=10,311)  - FOR/BUD (n=10,351)  - FOR/FLU (n=434)  - SAL/FLU (n=9,605)  - VIL/FLU (n=7,584) | |  |  |

COPD, chronic obstructive pulmonary disease; FDC, fixed-dose combinations; FOR/BEC, formoterol/beclomethasone; FOR/BUD, formoterol/budesonide; FOR/FLU, formoterol/fluticasone; ICS, Inhaled corticosteroids; IND/GLY, indacaterol/glycopyrronium; LABA, long-acting β_2_ agonists; LAMA, long-acting muscarinic antagonists; OLO/TIO, olodaterol/tiotropium; SAL/FLU, salmeterol/fluticasone; VIL/FLU, vilanterol/fluticasone; VIL/UME, vilanterol/umeclidinium.

**Figure S2.** Distributions of propensity score by study drug before and after PS matching

1. Before PS matching

**
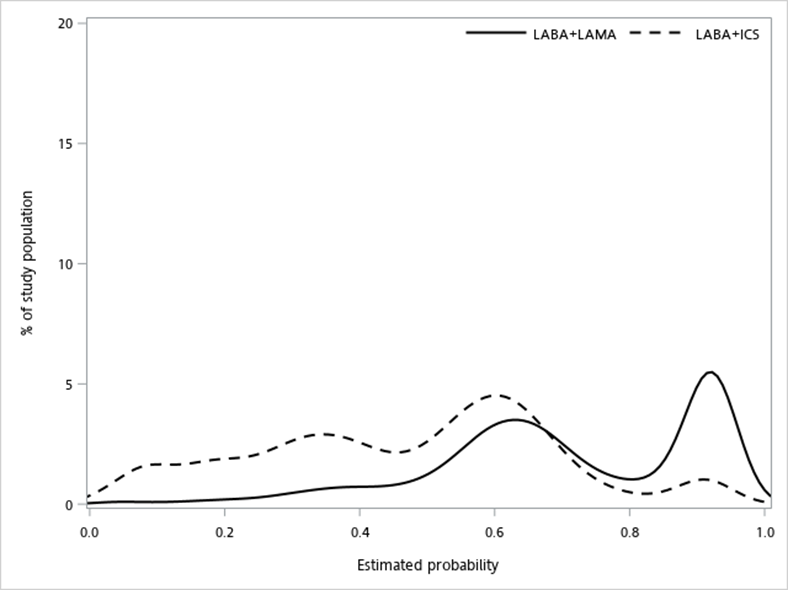
**

1.
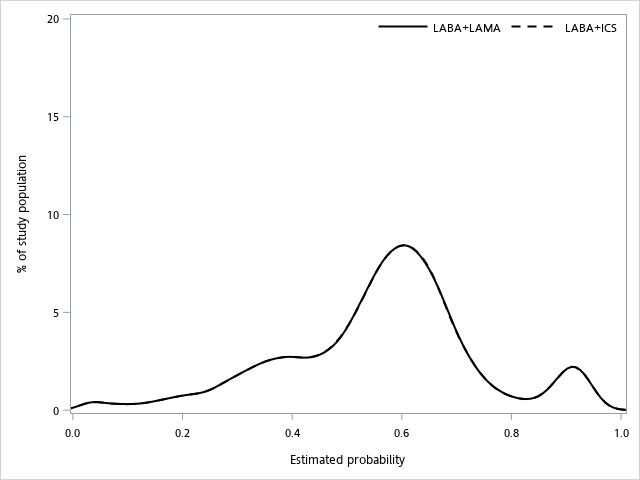
After PS matching^a^

FDC, fixed-dose combinations; ICS, inhaled corticosteroids; LABA, long-acting β_2_ agonists; LAMA, long-acting muscarinic antagonists; PS, propensity score.

^a^One LABA/LAMA FDC initiator versus one randomly sampled LABA/ICS FDC initiator in each matched subset.

**Figure S3.** Cumulative incidence plots of composite cardiovascular events by study FDC treatment before and after PS matching derived from complement of the Kaplan-Meier survival function

1. Before PS matching


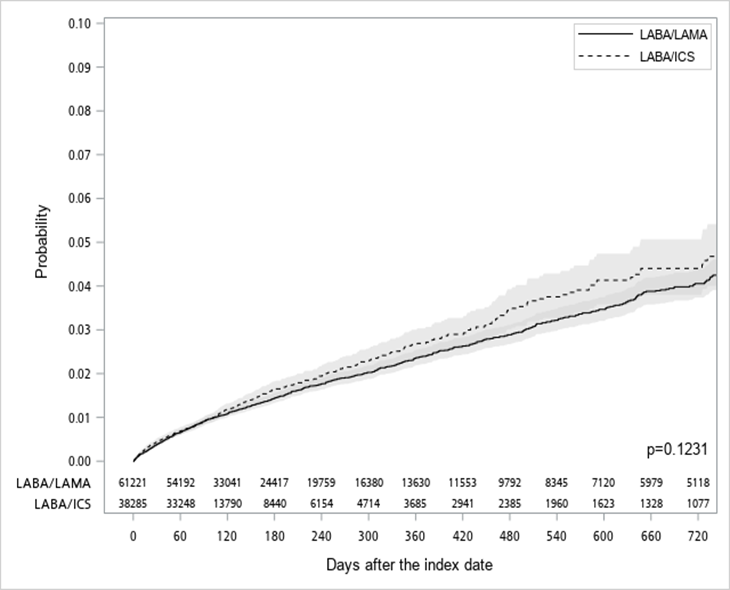


1. After PS matching^a^


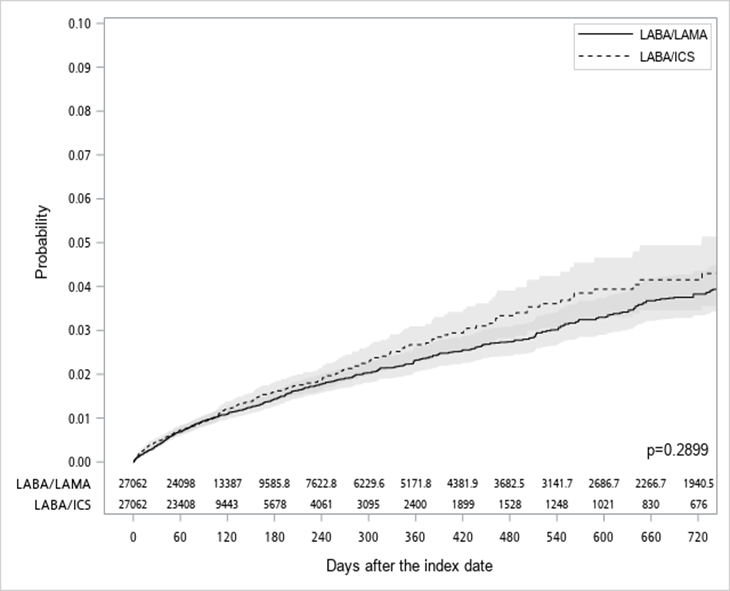


FDC, fixed-dose combinations; ICS, inhaled corticosteroids; LABA, long-acting β_2_ agonists; LAMA, long-acting muscarinic antagonists; PS, propensity score.

^a^Data after PS matching were weighted by the inverse of the matching ratio.

**Figure S4.** Cumulative incidence plots of composite cardiovascular events by study FDC treatment before and after PS matching accounting for the influence of competing risk from overall death

1. Before PS matching


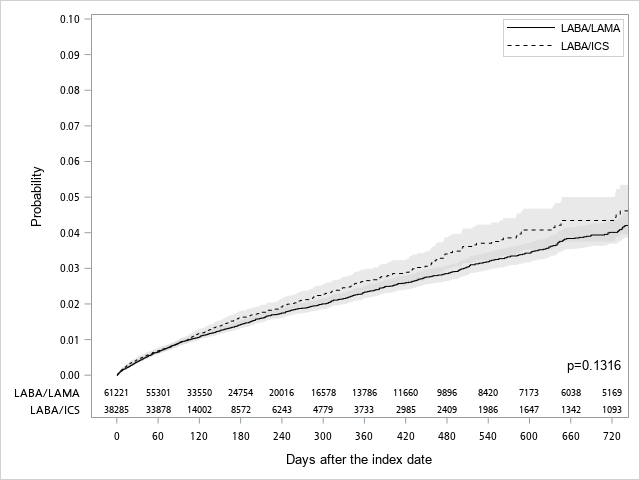


1. After PS matching^a^


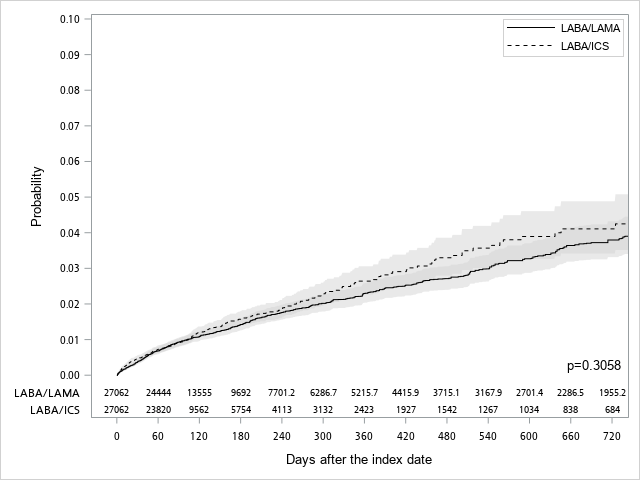


FDC, fixed-dose combinations; ICS, inhaled corticosteroids; LABA, long-acting β_2_ agonists; LAMA, long-acting muscarinic antagonists; PS, propensity score.

^a^Data after PS matching were weighted by the inverse of the matching ratio.

**eReferences**

1. National Health Insurance Administration. 2022-2023 National Health Insurance Annual Report. <https://www.nhi.gov.tw/Content_List.aspx?n=9223A12B5B31CB37&topn=4864A82710DE35ED> Accessed on February 26, 2023.
2. Lin LY, Warren-Gash C, Smeeth L, Chen PC. Data resource profile: the National Health Insurance Research Database (NHIRD). Epidemiol Health. 2018;40:e2018062.
3. Lee PC, Kao FY, Liang FW, Lee YC, Li ST, Lu TH. Existing data sources in clinical epidemiology: The Taiwan National Health Insurance Laboratory Databases. Clin Epidemiol. 2021;13:175-181.
4. National Health Insurance Administration. COPD pay-for-performance program [Chinese]. <https://www.nhi.gov.tw/Content_List.aspx?n=DDB1A5E178B7D09D&topn=5FE8C9FEAE863B46> Accessed on February 26, 2023.
5. Division of Pharmacoepidemiology and Pharmacoeconomics, Department of Medicine, Brigham and Women’s Hospital and Harvard Medical School, Boston, MA. The nearest-neighbor matching algorithm in the Pharmacoepidemiology Toolbox. <http://www.drugepi.org/dope-downloads/> Accessed on February 26, 2023.
6. Austin PC. An introduction to propensity score methods for reducing the effects of confounding in observational studies. Multivariate Behav Res. 2011;46(3):399-424.
7. Fine JP, Gray RJ. A proportional hazards model for the subdistribution of a competing risk. J Am Stat Assoc. 1999;94:496‐509.
8. Austin PC, Fine JP. Practical recommendations for reporting Fine-Gray model analyses for competing risk data. Stat Med. 2017;36(27):4391-4400.
9. Schneeweiss S, Rassen JA, Glynn RJ, Avorn J, Mogun H, Brookhart MA. High-dimensional propensity score adjustment in studies of treatment effects using health care claims data. Epidemiology. 2009;20(4):512-522.
10. Rassen JA, Blin P, Kloss S, Neugebauer RS, Platt RW, Pottegård A, et al. High-dimensional propensity scores for empirical covariate selection in secondary database studies: Planning, implementation, and reporting. Pharmacoepidemiol Drug Saf. 2023;32(2):93-106.
11. Granger E, Sergeant JC, Lunt M. Avoiding pitfalls when combining multiple imputation and propensity scores. Stat Med. 2019;38:5120-5132.
12. SAS Institute Inc. SAS/STAT® 14.1 User’s Guide The MI Procedure. <https://support.sas.com/documentation/onlinedoc/stat/141/mi.pdf> Accessed on February 26, 2023.
13. SAS Institute Inc. SAS/STAT® 13.1 User’s Guide The MIANALYZE Procedure. <https://support.sas.com/documentation/onlinedoc/stat/131/mianalyze.pdf> Accessed on February 26, 2023.
14. Dong YH, Chang CH, Lin JW, Yang WS, Wu LC, Toh S. Comparative cardiovascular effectiveness of glucagon-like peptide-1 receptor agonists versus sodium-glucose cotransporter-2 inhibitors in patients with type 2 diabetes: a population-based cohort study. Diabetes Obes Metab, 2022;24:1623-1637.
15. Sun SH, Chang CH, Zhan ZW, Chang WH, Chen YA, Dong YH. Risk of COPD exacerbations associated with statins versus fibrates: a new user, active comparison, and high-dimensional propensity score matched cohort study. Int J Chron Obstruct Pulmon Dis. 2021;16:2721-2733.
16. Cheng CL, Lee CH, Chen PS, Li YH, Lin SJ, Yang YH. Validation of acute myocardial infarction cases in the national health insurance research database in taiwan. J Epidemiol. 2014;24(6):500-507.
17. Coloma PM, Valkhoff VE, Mazzaglia G, Nielsson MS, Pedersen L, Molokhia M, et al. Identification of acute myocardial infarction from electronic healthcare records using different disease coding systems: a validation study in three European countries. BMJ Open. 2013;3(6):e002862.
18. Varas-Lorenzo C, Castellsague J, Stang MR, Tomas L, Aguado J, Perez-Gutthann S. Positive predictive value of ICD-9 codes 410 and 411 in the identification of cases of acute coronary syndromes in the Saskatchewan Hospital automated database. Pharmacoepidemiol Drug Saf. 2008;17(8):842-852.
19. Sundbøll J, Adelborg K, Munch T, Frøslev T, Sørensen HT, Bøtker HE, et al. Positive predictive value of cardiovascular diagnoses in the Danish National Patient Registry: a validation study. BMJ Open. 2016;6(11):e012832.
20. Cozzolino F, Montedori A, Abraha I, Eusebi P, Grisci C, Heymann AJ, et al. A diagnostic accuracy study validating cardiovascular ICD-9-CM codes in healthcare administrative databases. The Umbria Data-Value Project. PLoS One. 2019;14(7):e0218919.
21. Bosco-Lévy P, Duret S, Picard F, Dos Santos P, Puymirat E, Gilleron V, et al. Diagnostic accuracy of the International Classification of Diseases, Tenth Revision, codes of heart failure in an administrative database. Pharmacoepidemiol Drug Saf. 2019;28(2):194-200.
22. Hennessy S, Leonard CE, Freeman CP, Deo R, Newcomb C, Kimmel SE, et al. Validation of diagnostic codes for outpatient-originating sudden cardiac death and ventricular arrhythmia in Medicaid and Medicare claims data. Pharmacoepidemiol Drug Saf. 2010;19(6):555-562.
23. Hennessy S, Leonard CE, Newcomb C, Kimmel SE, Bilker WB. Cisapride and ventricular arrhythmia. Br J Clin Pharmacol. 2008;66(3):375-385.
24. Hsieh CY, Chen CH, Li CY, Lai ML. Validating the diagnosis of acute ischemic stroke in a National Health Insurance claims database. J Formos Med Assoc. 2015;114(3):254-259.
25. Hsieh MT, Hsieh CY, Tsai TT, Wang YC, Sung SF. Performance of ICD-10-CM diagnosis codes for identifying acute ischemic stroke in a National Health Insurance claims database. Clin Epidemiol. 2020;12:1007-1013.
26. Drahos J, Vanwormer JJ, Greenlee RT, Landgren O, Koshiol J. Accuracy of ICD-9-CM codes in identifying infections of pneumonia and herpes simplex virus in administrative data. Ann Epidemiol. 2013;23(5):291-293.
27. Levey AS, Stevens LA, Schmid CH, Zhang YL, Castro 3^rd^ AF, Feldman HI; CKD-EPI (Chronic Kidney Disease Epidemiology Collaboration). A new equation to estimate glomerular filtration rate. Ann Intern Med. 2009;150(9):604-612.
28. Rassen JA, Glynn RJ, Rothman KJ, Setoguchi S, Schneeweiss S. Applying propensity scores estimated in a full cohort to adjust for confounding in subgroup analyses. Pharmacoepidemiol Drug Saf. 2012;21(7):697-709.
29. Wang SV, Jin Y, Fireman B, Gruber S, He M, Wyss R, et al. Relative performance of propensity score matching strategies for subgroup analyses. Am J Epidemiol. 2018;187(8):1799-1807.
